# Origin of Short-Chain Organic Acids in Serpentinite Mud Volcanoes of the Mariana Convergent Margin

**DOI:** 10.3389/fmicb.2019.01729

**Published:** 2019-07-26

**Authors:** Philip Eickenbusch, Ken Takai, Olivier Sissman, Shino Suzuki, Catriona Menzies, Sanae Sakai, Pierre Sansjofre, Eiji Tasumi, Stefano M. Bernasconi, Clemens Glombitza, Bo Barker Jørgensen, Yuki Morono, Mark Alexander Lever

**Affiliations:** ^1^Institute of Biogeochemistry and Pollutant Dynamics, ETH Zürich, Zurich, Switzerland; ^2^SUGAR Program, Institute for Extra-Cutting-Edge Science and Technology Avant-Garde Research (X-star), Japan Agency for Marine-Earth Science Technology, Yokosuka, Japan; ^3^IFP Energies Nouvelles, Rueil-Malmaison, France; ^4^Geomicrobiology Research Group, Kochi Institute for Core Sample Research, Japan Agency for Marine-Earth Science and Technology, Kochi, Japan; ^5^Ocean and Earth Science, National Oceanography Centre, University of Southampton, Southampton, United Kingdom; ^6^Department of Geology and Petroleum Geology, University of Aberdeen, Aberdeen, United Kingdom; ^7^Laboratoire Géosciences Océan UMR 6538, Université de Bretagne Occidentale, Brest, France; ^8^Geological Institute, ETH Zürich, Zurich, Switzerland; ^9^NASA Ames Research Center, Moffett Field, CA, United States; ^10^Department of Bioscience, Center for Geomicrobiology, Aarhus University, Aarhus, Denmark

**Keywords:** limits of life, deep biosphere, serpentinization, abiotic synthesis, formate, acetate, methane, International Ocean Discovery Program

## Abstract

Serpentinitic systems are potential habitats for microbial life due to frequently high concentrations of microbial energy substrates, such as hydrogen (H_2_), methane (CH_4_), and short-chain organic acids (SCOAs). Yet, many serpentinitic systems are also physiologically challenging environments due to highly alkaline conditions (pH > 10) and elevated temperatures (>80°C). To elucidate the possibility of microbial life in deep serpentinitic crustal environments, International Ocean Discovery Program (IODP) Expedition 366 drilled into the Yinazao, Fantangisña, and Asùt Tesoru serpentinite mud volcanoes on the Mariana Forearc. These mud volcanoes differ in temperature (80, 150, 250°C, respectively) of the underlying subducting slab, and in the porewater pH (11.0, 11.2, 12.5, respectively) of the serpentinite mud. Increases in formate and acetate concentrations across the three mud volcanoes, which are positively correlated with temperature in the subducting slab and coincide with strong increases in H_2_ concentrations, indicate a serpentinization-related origin. Thermodynamic calculations suggest that formate is produced by equilibrium reactions with dissolved inorganic carbon (DIC) + H_2_, and that equilibration continues during fluid ascent at temperatures below 80°C. By contrast, the mechanism(s) of acetate production are not clear. Besides formate, acetate, and H_2_ data, we present concentrations of other SCOAs, methane, carbon monoxide, and sulfate, δ^13^C-data on bulk carbon pools, and microbial cell counts. Even though calculations indicate a wide range of microbial catabolic reactions to be thermodynamically favorable, concentration profiles of potential energy substrates, and very low cell numbers suggest that microbial life is scarce or absent. We discuss the potential roles of temperature, pH, pressure, and dispersal in limiting the occurrence of microbial life in deep serpentinitic environments.

## Introduction

Since the 1950s, advances in sampling techniques have extended explorations of subseafloor life from ~8 meters below seafloor (mbsf) (Morita and Zobell, 1955) to ~2,500 mbsf (Inagaki et al., 2015). Today, the subseafloor microbial biosphere is estimated to account for 0.18–3.6% of total living biomass on Earth (Kallmeyer et al., 2012), and to persist in many places despite low energy supply and harsh environmental conditions, such as high temperature, pressure, salinity, and/or pH (Takai, 2011; Hoehler and Jørgensen, 2013; Lever et al., 2015). These variables affect the power requirements of microbial life, e.g., by increasing rates of biomolecule damage, and consequently also raise the power required by cells to maintain and repair essential biomolecules (Lever et al., 2015). As a result, the presence and abundance of microorganisms within subseafloor habitats varies greatly with location as a result of stark differences in cell-specific power supply and cell-specific power demand (Inagaki et al., 2015; Lever et al., 2015; Møller et al., 2018; Heuer et al., 2019).

In subseafloor sediments, most microorganisms are chemoorganotrophic and rely on the breakdown of photosynthetically fixed organic matter (OM) and *in situ*-produced microbial necromass as power sources (Canfield et al., 2005; Lomstein et al., 2012). Under anaerobic conditions, SCOAs, such as formate, acetate, propionate, butyrate, and lactate, in addition to H_2_, are important metabolic intermediates, being the end products of microbial fermentation and acetogenesis reactions (Stams, 1994; Wellsbury et al., 2002; Worm et al., 2010). In addition, SCOAs are key energy substrates for microorganisms involved in terminal oxidation reactions to carbon dioxide (CO_2_) and CH_4_ involving nitrate, manganese(IV), iron(III), sulfate, and CO_2_ as electron acceptors (Froelich et al., 1979; Sørensen et al., 1981; Canfield et al., 1993; Finke and Jørgensen, 2008). Microbial production and turnover of SCOAs has been reported from 0 (Finke et al., 2007) to 80°C (Wellsbury et al., 1997; Parkes et al., 2007), and pH values of <4 (Goodwin and Zeikus, 1987; Koschorreck, 2008) to >12 (Yu et al., 2013), and to sediment depths of ~800 mbsf (Wellsbury et al., 2002).

In addition to being degraded by microorganisms, photosynthetically fixed OM, and microbial necromass can be broken down at high temperature by thermochemical or “thermogenic” reactions (Wellsbury et al., 1997; Egeberg and Barth, 1998). Elevated temperatures in petroleum and gas reservoirs, hydrothermal sediments, or deeply buried sediment layers produce many of the same intermediates and end products released during the microbial breakdown of OM, including SCOAs, H_2_, and CH_4_ (Wellsbury et al., 1997; Parkes et al., 2007). Field and laboratory experiments show that thermogenic SCOA pools are typically dominated by acetate (e.g., Cooles et al., 1987; Lundegard and Kharaka, 1990; Barth and Bjørlykke, 1993; Kharaka et al., 1993; Shebl and Surdam, 1996), and in exceptional cases by propionate (Carothers and Kharaka, 1978). Furthermore, in thermogenic environments with temperatures >100°C in the presence of sulfate, significant fractions of the SCOAs, H_2_, and hydrocarbons produced by thermogenic breakdown of OM can be removed through thermochemical sulfate reduction (e.g., Mottl et al., 1979; Kiyosu and Krouse, 1990; Worden et al., 2000; Cross et al., 2004; Truche et al., 2009).

In addition to the breakdown of photosynthetically fixed and necromass bound OM, SCOAs can be synthesized via the abiotic reduction of inorganic carbon with electron donors released by serpentinization reactions (Holm and Andersson, 1998; McCollom and Seewald, 2007; Schrenk et al., 2013; Früh-Green et al., 2014; Preiner et al., 2018). Serpentinization reactions include chemical reactions whereby water reacts with ultramafic rocks rich in iron(II)-containing minerals, such as olivine and pyroxene, to release H_2_ (McCollom and Bach, 2009; Preiner et al., 2018). This H_2_ can then reduce inorganic carbon to formate, with which it forms a metastable equilibrium between 175 and 300° (McCollom and Seewald, 2001, 2003a). Mineral surface-catalyzed Sabatier-type and Fischer-Tropsch type reactions can furthermore cause H_2_ to react abiotically with CO or CO_2_ to form methanol, CH_4_, and SCOAs and hydrocarbons with ≥2 C atoms (Sabatier and Senderens, 1899; Fischer and Tropsch, 1926; McCollom and Seewald, 2003a, 2007; Holm and Neubeck, 2009). Rates of serpentinization and FTT reactions strongly depend on temperature, availability of suitable catalysts, and H_2_ and CO partial pressures (Van Der Laan and Beenackers, 1999), with H_2_ release peaking at ~300°C and CH_4_ production peaking at 320°C in batch experiments ranging from 200 to 320°C (McCollom et al., 2016). Importantly, while the production of CH_4_ and hydrocarbons with ≥2 C atoms by FTT in the presence of a gas phase is generally accepted, recent studies have challenged earlier reports of CH_4_ production by FTT in water-saturated serpentinitic systems at 200–300°C (McDermott et al., 2015; McCollom, 2016; Wang et al., 2018). Instead stable and clumped isotopic compositions point toward synthesis of these hydrocarbons at ≥400°C from magmatic volatiles that are trapped within fluid-vapor inclusions. Alteration of mantle rocks by serpentinization then releases these compounds into circulating fluids.

A typical indicator of serpentinization reactions is the presence of fluids with pH >10 and high formate concentrations (Mottl et al., 2003; McCollom and Bach, 2009; Schrenk et al., 2013). In some places, large macrofaunal populations are present where serpentinite-influenced fluids mix with seawater at the seafloor (Kelley et al., 2005; Fryer, 2012; Ohara et al., 2012; Joseph, 2017). These macrofauna feed on abundant chemotrophic microbiota which are sustained by abiotically produced H_2_, CH_4_, and SCOAs as energy donors and seawater-derived O_2_ and sulfate as electron acceptors. It has been suggested that the same abiotically produced compounds might also support microbial communities in the upper mantle and in subseafloor basaltic ocean crust (Früh-Green et al., 2004; Lever et al., 2013; Bach, 2016; Smith et al., 2019).

Well-known sites of serpentinization in the marine environment include the Lost City Hydrothermal Field (LCHF; Früh-Green et al., 2003, 2014; Kelley et al., 2005; Proskurowski et al., 2008; Konn et al., 2009; Lang et al., 2010, 2018) and the Mariana Forearc (Fryer et al., 2003, 2018b; Mottl et al., 2003; Hulme et al., 2010; Fryer, 2012). The LCHF is located on the Atlantis Massif, an oceanic core complex, 15 km west of the Mid-Atlantic Ridge and is a model environment for rock-hosted serpentinitic marine systems. In contrast, serpentinite mud volcanoes of the Mariana forearc are located in a subduction zone. Here, highly alkaline serpentinite muds, produced >10 kmbsf in the mantle wedge by alteration of mantle rock with fluids liberated from the underlying subducting plate, are transported to the seafloor (Hulme et al., 2010; Fryer, 2012). Geochemical profiles and membrane lipids in muds of the top 20 mbsf indicate the presence of metabolically active microbial life that is dominated by Archaea (Mottl et al., 2003; Curtis et al., 2013; Aoyama et al., 2018; Kawagucci et al., 2018). Comparing 16S rRNA gene sequences of Curtis et al. (2013) with published sequences (M. Lever, *unpubl*.) indicates the presence of Lokiarchaeota with close relatives in methane hydrates, methane seeps, and anoxic subseafloor sediments, and of aerobic nitrifying Marine Group I Thaumarchaeota with close relatives in diverse deep sea benthic surface habitats. Cultivation studies have resulted in the isolation of alkaliphilic bacteria (*Marinobacter alkaliphilus*), which grow at a pH of 6.5 to 10.8–11.4 using organic substrates as electron donors, and O_2_, nitrate, or fumarate as electron acceptors (Takai et al., 2005).

Distinguishing microbial, thermogenic, and abiotic origins of SCOAs remains a challenge in many locations because these processes overlap in temperature, pressure, and redox ranges and can, in theory, co-occur in the same environments (Holm and Andersson, 1998; Schrenk et al., 2013). Furthermore, as mentioned earlier, microbially, thermogenically, and abiotically produced SCOAs can also be removed by microbial, thermogenic, and abiotic processes. Thus, accumulation of SCOAs is not a reliable indicator of origin. Even if microbial, thermogenic, and abiotic processes are spatially separated, fluids may contain products of all three processes if they are mixtures with multiple origins. Fortunately, in some cases SCOA origin can be revealed by concentration, isotopic, and thermodynamic analyses of natural educts and products (Heuer et al., 2009; Lang et al., 2010, 2018; Lever et al., 2010).

Here we investigate the origin of SCOAs in three serpentinite mud volcanoes (Yinazao, Fantangisña, and Asùt Tesoru) of the Mariana forearc system based on mud and extracted pore fluids that were obtained during IODP Expedition 366 in 2016/2017. We compare depth-related trends in SCOA concentrations to those observed for other microbial energy substrates (H_2_, CO, CH_4_), and electron acceptors (${\text{SO}}_{4}^{2-}$, DIC), to stable isotopic compositions of bulk carbon pools [DIC, total organic carbon (TOC), dissolved organic carbon (DOC)], and to cell counts. Furthermore, we present results of incubation experiments and of thermodynamic calculations to elucidate likely SCOA sources. Despite high concentrations of SCOAs and other microbial substrates and low temperatures of samples (<10°C), microbial activity and microbial populations are around the detection limit. This (near-)absence of active microbial populations is likely due to elevated temperature (~80–250°C) in the slab and/or high pH (pH 11.0–12.5) of serpentinite mud, and due to absence of significant microbial colonization of serpentinite mud during ascent from the mantle wedge to the seafloor. Due to the absence of a clear microbial imprint, the mud fluids sampled during Expedition 366 provide a unique window into the reactions that produce microbial energy substrates deep within subduction zones.

## Materials and Methods

### Site Description

**Figure 1:**
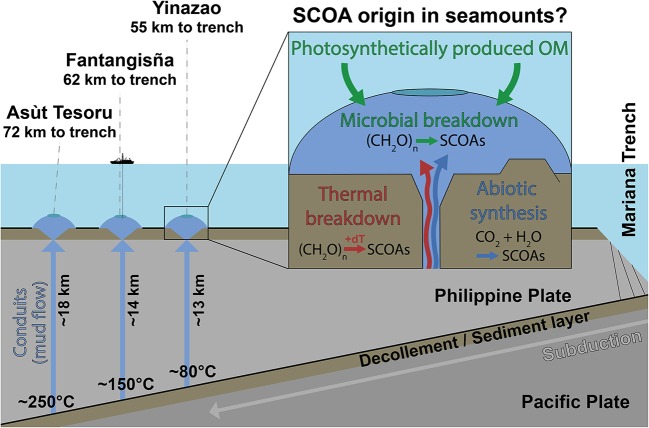
Schematic dissection of the Yinazao, Fantangisña, and Asùt Tesoru mud volcanoes in relation to the Mariana Trench, which is formed by subduction of the Pacific Plate under the Philippine Plate. The Pacific Plate and its sediment cover are exposed to increasing temperatures as they are subducted. The Yinazao, Fantangisña, and Asùt Tesoru mud volcanoes are shown in blue due to the distinct blue color of the dominant lithology, upwelling serpentinite mud. Investigating how SCOA compositions, and origins (microbial, thermogenic, abiotic) change both horizonally and vertically between and within these mud volcanoes provides insights into the controls on carbon transformation reactions in the deep subseafloor.

IODP Expedition 366 (8 December 2016 to 7 February 2017) onboard the *R/V JOIDES Resolution* drilled into the Yinazao (previously known as Blue Moon), Fantangisña (previously known as Celestial), and Asùt Tesoru (previously known as Big Blue) serpentinite mud volcanoes on the Mariana forearc (Fryer et al., 2018b). These mud volcanoes are located to the west of the Mariana Trench and differ in distance to the trench and temperature of the underlying subducting slab (Figure 1). The three mud volcanoes also differ in geographic location, i.e., Fantagisña is located ~90 km north of Yinazao, and ~170 km south of Asùt Tesoru (Fryer et al., 2018b). Yet, distance to trench, which only differs by 17 km, and associated changes in the slab temperature, which varies from 80°C to around 250°C, are more important drivers of deep geochemical processes (Hulme et al., 2010). For this reason we schematically represent the three mud volcanoes as a transect in Figure 1.

All three mud volcanoes are formed as fluids, liberated during the subduction of both sediments and crustal rock from the Pacific plate, hydrate the overlying plate's mantle and drive serpentinization, whereby mud is produced by rock-alteration and breakdown, and the pH of fluids becomes highly alkaline (Mottl et al., 2003; Fryer, 2012). Due to volume expansion and corresponding density changes, this mud wells up via conduits, which are likely related to fault intersections, to the seafloor (Früh-Green et al., 2004), where it forms serpentinite mud volcanoes with heights and diameters of multiple kilometers (Fryer et al., 2018b). Maximum vertical mud flow velocities at Yinazao and Asùt Tesoru have been estimated to be 10.3 and 36.3 cm yr^−1^, respectively (Hulme et al., 2010; no data for Fantangisña), which correspond to ascent times of ~130,000 (Yinazao) and ~50,000 years (Asùt Tesoru) from the décollement to the seafloor. Detailed site data from IODP Expedition 366 are publicly available on the IODP homepage (Fryer et al., 2018b).

### Sampling

Table 1 provides an overview of drilled sites included in this study. Most samples were obtained using a Half-Length Advanced Piston Corer. Site U1492C Core 1 was obtained by a full-length Advanced Piston Corer. Site U1498 samples were obtained using a Rotary Core Barrel. Based on estimated temperature gradients for all flank and summit sites, *in situ* temperatures of all cores were <10°C. For further details, we refer to Fryer et al. (2018a).

**Table 1:** Background information on drilled holes during IODP Expedition 366.

**Hole**	**Seamount**	**Location**	**Latitude**	**Longitude**	**Water depth (mbsl)**	**Coring**	**Total penetration (m)**	**Distance to trench (km)**	**Distance to slab (km)**	**Temperature at slab (°C)**
U1492C	Yinazao	Summit	15°42.5590′N	147°10.6001′E	3666.47	APC/HLAPC	139.1	55	13	~80
U1493B	Asùt Tesoru	Flank	17°59.1665′N	147°06.0060′E	3358.92	HLAPC	32.6	72	18	~250
U1494A	Asùt Tesoru	Flank	18°3.0896′N	147°6.0003′E	2199.80	HLAPC	39	72	18	~250
U1495A	Asùt Tesoru	Flank	18°05.6693′N	147°06.0004′E	1405.81	HLAPC	10.7	72	18	~250
U1495B	Asùt Tesoru	Flank	18°05.6788′N	147°05.9901′E	1401.89	HLAPC	10.8	72	18	~250
U1496A	Asùt Tesoru	Summit	18°6.5936′N	147°6.0999′E	1243.38	HLAPC	44.8	72	18	~250
U1496B	Asùt Tesoru	Summit	18°6.6205′N	147°6.0998′E	1240.18	HLAPC	36	72	18	~250
U1497A	Fantangisña	Summit	16°32.2536′N	147°13.2642′E	2019.24	HLAPC	34.2	62	14	~150
U1497B	Fantangisña	Summit	16°32.2528′N	147°13.2606′E	2018.22	HLAPC	23.8	62	14	~150
U1498A	Fantangisña	Flank	16°27.0898′N	147°09.8502′E	3496.21	RCB	181.6	62	14	~150
U1498B	Fantangisña	Flank	16°27.3716′N	147°10.1166′E	3284.70	RCB	260	62	14	~150

*All data from Fryer et al. (2018c), except distance to trench (from Hulme et al., 2010), and distance to slab data (Oakley, 2008; from Oakley et al., 2007, 2008)*.

### Porewater

Interstitial water samples were extracted by squeezing water out of inner parts of cores using Carver presses (Manheim, 1966) with filtering through prewashed 11 μm cellulose filters (Whatman, Cat.-No. 1001090). Water samples were then filtered through 0.45 μm PES syringe filters (GE Puradisc, Cat-No. 6780.2504) during splitting into aliquots. pH, and concentrations of [Mg_(aq)_], sulfate, DIC and DOC were determined onboard as described in Fryer et al. (2018a). All data are available online (Fryer et al., 2018b,c).

#### SCOA Quantification

Porewater samples for SCOA quantification were stored at −80°C in baked vials (6 h at 450°C) immediately after retrieval and were quantified in the home laboratory using two-dimensional ion chromatography as described in Glombitza et al. (2014).

#### DIC and DOC Concentrations

DIC and DOC concentrations were measured onboard with the OI Analytical Aurora 1030C TOC analyzer, consisting of a syringe module, a sample-stripping manifold, and an infrared CO_2_ analyzer. Porewater samples (1 mL per injection) were acidified with 0.2 mL of 2 M HCl. The CO_2_ released during this acid addition step was stripped and injected into the CO_2_ analyzer. Subsequently, any remaining carbon in the sample was combusted, and the DOC was obtained by difference. The CO_2_ Beer-Lambert absorption law was integrated to determine the total CO_2_ released from the sample (Fryer et al., 2018a).

#### δ^13^C-DOC Values

Porewater samples for δ^13^C-DOC analyses were stored at −80°C in baked vials (6 h at 450°C). Isotopic compositions were measured after wet-chemical oxidation of DOC with persulfate (1 h at 100°C) on decarbonized subsamples (acidification to pH < 3 with 85% H_3_PO_4_). Two to four ml of headspace were then transferred into He flushed vials and analyzed using isotope-ratio mass spectrometry as described in Lang et al. (2012).

#### δ^13^C-DIC Values

Porewater samples for δ^13^C-DIC analyses were preserved by adding HgCl_2_ and stored at 4°C. Depending on the concentration, 1–2 ml of sample were injected into He-flushed exetainers containing 150 μl of 85% phosphoric acid to lower the pH and convert all DIC into CO_2_. CO_2_ was then measured after equilibration using isotope-ratio mass spectrometry. Standardization was accomplished by measuring Na-bicarbonate solutions of different concentrations prepared from a Na-bicarbonate powder, for which the δ^13^*C* was determined by digestion with phosporic acid, and by comparison to calcium carbonate standards as described in Breitenbach and Bernasconi (2011).

### Serpentinite Mud

#### Cell Counts

To determine cell abundances, 2 cm^3^ of mud were subsampled from central portions of cores using sterile cut-off syringes in an ultra clean air environment (KOACH T-500f, Koken, Ltd., Morono et al., 2018). Samples were immediately fixed with 2% paraformaldehyde in 3-(cyclohexylamino)-1-propanesulfonic acid (CAPS) buffer solution adjusted to pH 11. The fixed samples were stored at 4°C until the analysis at the home laboratory, whereby slurry samples were subjected to cell detachment and separation steps at the super clean room in Kochi Core Center, Japan (Morono et al., 2013, 2017). In brief, 1 mL of fixed mud slurry was mixed with 1.4 mL of 2.5% NaCl, 300 μL of detergent mix (100 mM ethylenediamine tetra-acetic acid [EDTA], 100 mM sodium pyrophosphate, 1% [v/v] Tween-80), and 300 μL of pure methanol, and homogenized on a Shake Master (Bio Medical Science, Japan) at 500 rpm for 60 min. Samples were then sonicated at 160 W for 30 s for 10 cycles (Bioruptor UCD-250HSA; Cosmo Bio, Japan), followed by loading onto density layers composed of 30% Nycodenz (1.15 g/cm^3^), 50% Nycodenz (1.25 g/cm^3^), 80% Nycodenz (1.42 g/cm^3^), and 67% sodium polytungstate (2.08 g/cm^3^), and centrifugation at 10,000 × *g* for 1 h at 25°C with swinging rotors. The light density layer was collected, whereas the heavy fraction was subjected to a second round of separation after washing with 5 mL of 2.5% NaCl. The recovered supernatants were then pooled and passed through a 0.22 μm polycarbonate membrane filter. Cells on the membrane filter were stained with SYBR Green I staining solution (1/40 of SYBR Green I in Tris-EDTA [TE] buffer). The number of SYBR Green I–stained cells was enumerated by automated epifluorescence microscopic counting (Morono et al., 2009; Inagaki et al., 2015). To quantify procedural contamination, blank controls involving 1 mL of 2.5% NaCl solution were also subjected to the above cell separation and staining procedures. The procedural contamination was on average 1.0 cells per counted membrane (*n* = 10), which corresponds to a minimum quantification limit of 24 cells/cm^3^ (average plus three times the standard deviation of blank counts).

#### Gas Analyses

To quantify concentrations of H_2_, CH_4_, and CO, 1 cm^3^ of mud was collected from cut ends of core sections immediately after core arrival. Samples were placed in 20 cm^3^ glass vials with 3 mL of distilled water and a small amount of HgCl_2_ to prevent microbial activity. Vials were sealed with Teflon-coated butyl rubber septa and crimped aluminum caps and then placed in an oven at 80°C for 30 min. A 0.5 cm^3^ aliquot of the headspace was sampled with a standard gas syringe and automatically injected into a GL Science GC4000 GC equipped with a helium ionization detector set at 250°C. The column (2 mm inner diameter; 6.3 mm outer diameter) was packed with carbosieve (Agilent/Hewlett Packard). The GC oven program was set to 40°C during the initial 5 min with a subsequent rise to 250°C at 20°C/min. A second 0.5 cm^3^ aliquot of the headspace was then automatically injected into an Agilent/Hewlett Packard 6890 Series II gas chromatograph (GC) equipped with a flame ionization detector set at 250°C. The column (2 mm inner diameter; 6.3 mm outer diameter) was packed with 80/100 mesh HayeSep (Restek). The GC oven program was set to 80°C for 8.25 min with a subsequent rise to 150°C at 40°C/min. All measurements were calibrated using two different gas standards.

#### Total Organic Carbon (TOC) and δ^13^C-TOC

Analyses were performed on solid residue samples after porewater squeezing. TOC and δ^13^C_org_ were analyzed using an elemental analyzer (EA, Flash 2000; Thermo Scientific) coupled to a Delta V+ isotope ratio mass spectrometer (Thermo Scientific) at the Pôle de Spectrométrie Océan (PSO, Brest, France). Approximately 25 mg of decarbonated samples were loaded into tin capsules and introduced into an autosampler. Flash combustion was performed using an 8 s injection time of dioxygen at a flux of 240 mL min^−1^. Carbon isotope ratios were obtained against reference standards (SED-IVA reference number: 33802151) and in-house standards (Acetanilide: reference number 274462 from Thermo Fisher; CAP (leaf litter) and LIPG (yeast) from Institut de Physique du Globe de Paris, France). δ^13^C-TOC values are given as the per mil (‰) difference from the PDB standard. TOC was measured using the thermal conductivity detector of the Flash 2000 instrument. Routine replicate measurements had internal deviations of 0.15‰ for δ^13^C-TOC and <5% for TOC.

### Thermodynamic Calculations

Gibbs energy yields (Δ*G*_*r*_*)* of potential microbial, thermogenic, and abiotic reactions were calculated based on the equation

(1) $$\Delta G_{r}=\Delta G_{r}^{0}{\;}_{\left(T,p\right)}+RT\;ln\;Q_{r}$$

where $\Delta G_{r}^{0}{}_{\left(T,p\right)}$ is the Gibbs energy (kJ mol^−1^ of reaction) at standard concentrations (1 M per each reactant and product, pH 7.0) corrected for *in situ* temperature *T* (K) and pressure *p* (bar), *R* is the universal gas constant (0.008314 kJ mol^−1^ K^−1^), and *Q*_*r*_ the quotient of product and reactant activities. To obtain $\Delta G_{r}^{0}{}_{(T,p),}$ the standard Gibbs energies of the reaction $\Delta G_{r}^{0}$ was corrected to estimated *in situ* slab temperature and pressure as outlined in Stumm and Morgan (1996). Standard Gibbs energies, standard enthalpies, and standard molal volumes of formation are shown in Supplementary Table 1. Calculations were done for activities of aqueous species, which were calculated using the activity coefficients γ_CO32−_ = 0.038 (Plummer and Sundquist, 1982), γ_CH4_ = 1.24 (Millero, 2000), γ_SO42−_ = 0.104 (Millero and Schreiber, 1982), and γ_HS−_ = 0.685 (Clegg and Whitfield, 1991). The activity coefficients of H_2_ and CO were approximated with that of CH_4_, those of SCOAs were approximated with that of HS^−^, and those of glucose were set to 1.0. All concentrations were measured, except for glucose and HS^−^, which were assumed to equal 1 nM and 1 mM, respectively. The Δ*G*_*r*_ was calculated for temperature, pressure, and pH in sediment cores and for temperature, pressure, and pH under slab conditions. The pH under slab conditions was 11.2 for Yinazao, 11.0 for Fantangisña, and 12.5 for Asùt Tesoru (based on Fryer et al., 2018b).

## Results

**Figure 2:**
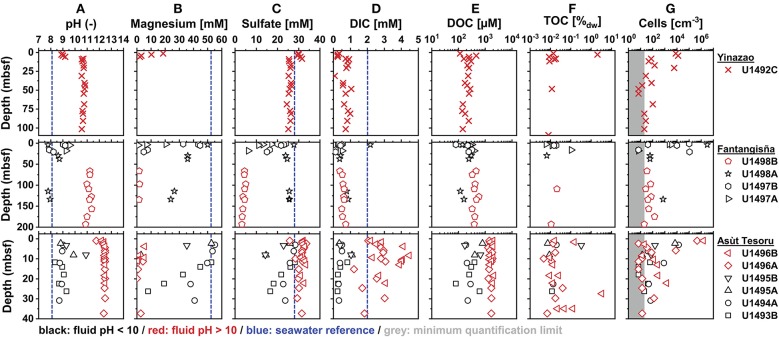
Depth profiles of **(A)** pH, **(B)** magnesium (Mg^2+^), **(C)** sulfate, **(D)** DIC, **(E)** DOC, **(F)** TOC, and **(G)** cell counts ordered in ascending distance to the Mariana Trench (top to bottom). All pH data, and magnesium (Mg^2+^), sulfate, DIC, and DOC concentrations were measured shipboard and were obtained from Fryer et al. (2018b). TOC (% sample dry weight) and cell abundances are from this study (gray area indicates counts that are below the quantification limit of 20 cells cm^−3^). Blue marks on the x-axis indicate seawater concentrations from Mottl et al. (2003). Red symbols indicate high-pH holes, black symbols indicate moderate-pH holes (see text for definitions).

**Figure 3:**
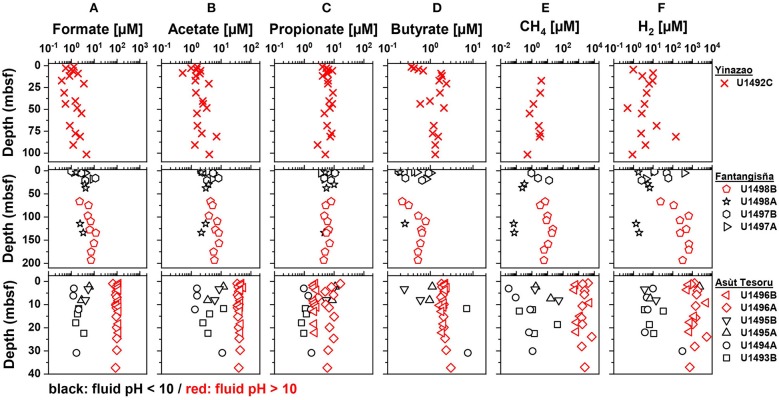
Depth profiles of **(A)** formate, **(B)** acetate, **(C)** propionate, **(D)** butyrate, **(E)** CH4, and **(F)** H2 concentrations in the Yinazao, Fantangisña, and Asùt Tesoru mud volcanoes ordered in ascending distance to the Mariana Trench (top to bottom). Red symbols indicate high-pH holes with an average pH > 10, black symbols indicate moderate-pH holes with an average pH < 10.

**Figure 4:**
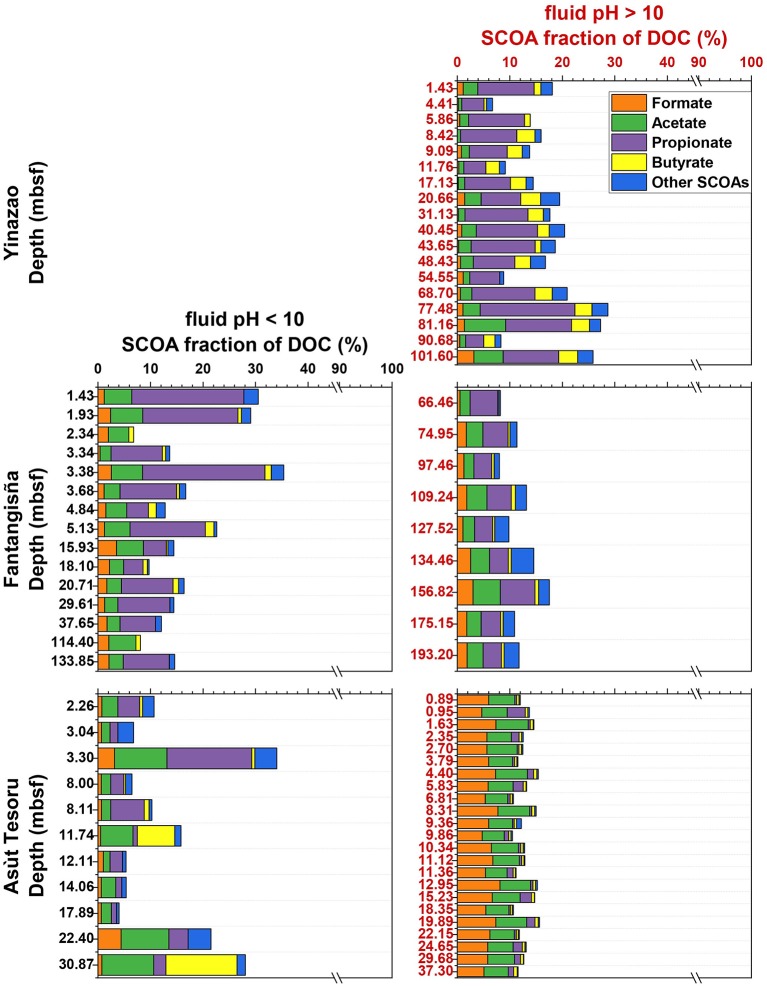
SCOA percent fractions of DOC vs. depth at the Yinazao, Fantangisña, and Asùt Tesoru mud volcanoes. Black labeling indicates moderate pH fluids with an average pH < 10 **(Left)**, red labeling indicates high pH fluids with an average pH > 10 **(Right)**.

**Figure 5:**
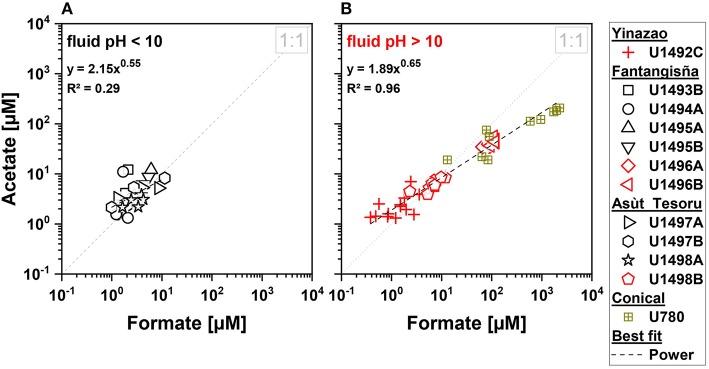
Formate-to-acetate ratios for three serpentinite mud volcanoes drilled during IODP Expedition 366. Black symbols indicate moderate pH fluids with an average pH < 10 **(A)**, and red symbols indicate high pH fluids with an average pH > 10 **(B)**. Brown symbols indicate data on high pH fluids from Conical seamount from ODP Leg 125 (Haggerty and Fisher, 1992).

**Figure 6:**
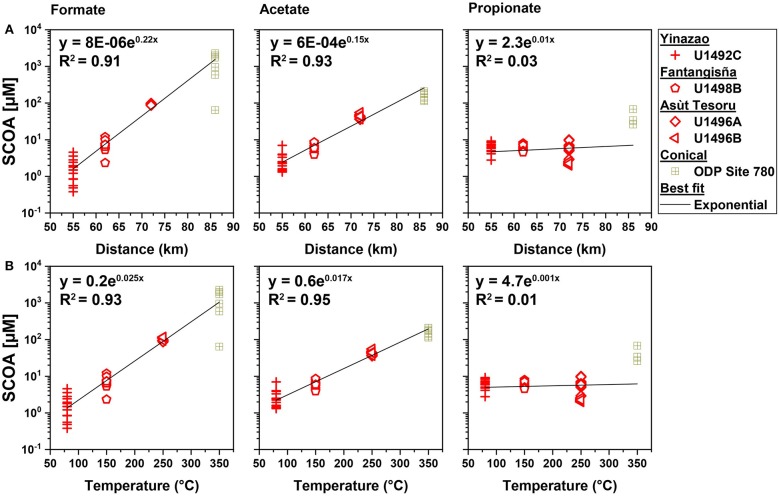
Formate, acetate and propionate concentrations in high pH fluids vs. distance to trench **(A)** and vs. slab temperatures **(B)**. Regression includes data from Conical seamount, which was drilled during Ocean Drilling Program Leg 125 (Haggerty and Fisher, 1992).

**Figure 7:**
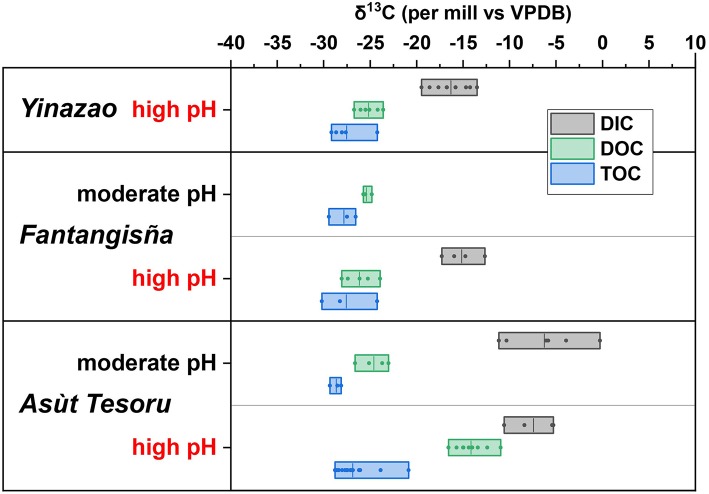
Box plots of δ^13^C-isotopic compositions of TOC, DOC, and DIC of moderate-pH and high-pH subsurface mud fluids. Boxes show the entire data range, vertical lines within boxes indicate the mean value.

In the following sections we first compare geochemical background data (pH; magnesium, sulfate, DIC, and DOC concentrations; TOC contents) and cell counts on high-pH subsurface serpentinite mud fluids (pH 10.5–12.5) with shallow and adjacent samples, that are under stronger seawater or sedimentary influence and have moderate pH values (pH 7.8–10; Figure 2). We then examine how concentration profiles of potential microbial energy substrates (SCOAs, CH_4_, H_2_, CO; Figure 3) vary with pH within and across the three volcanoes. In the final part, we investigate how the relative contributions of different SCOAs to DOC change between and within mud volcanoes (Figure 4), how the concentrations of individual SCOAs change in high-pH samples with distance to the trench and with increasing slab temperature (Figures 5, 6), and how δ^13^C*-*isotopic values of DIC, DOC, and TOC change between and within mud volcanoes (Figure 7). Through this integrated analysis, we provide insights into the role of deep, tectonically driven reactions in determining the sources and chemical compositions of microbial energy substrates in mud volcanoes of the Mariana forearc.

### Geochemical Setting

Porewater pH values increase downward from the seafloor (Figure 2A), where values approach those of seawater (pH 8.1). In samples with high fluid upflow, the pH stabilizes at values that increase with distance to trench (Yinazao: ~10.7; Fantangisña: ~11.2; Asùt Tesoru: ~12.4) and at different sediment depths (Yinazao: ~8 mbsf; Fantangisña: unclear but within top 66 mbsf; Asùt Tesoru: ~4 mbsf). Within Fantangisña and Asùt Tesoru, fluids from flank sites or areas with less fluid upflow are clearly distinguishable from fluids in areas of higher upflow based on pH values closer to those in surface sediments (Mottl et al., 2003). For the sake of simplicity, we from now on refer to samples from boreholes with high upflow and pH>10.0 as “high-pH mud fluids,” and samples from boreholes on flank sites and sites with less upflow and pH<10.0 as “moderate-pH mud fluids.” We, moreover, distinguish between surface samples with a clear seawater influence, and subsurface samples, where pH values are constant at higher values.

Magnesium (Mg^2+^)concentrations are mostly below or close to the detection limit of 0.1 mM in subsurface high-pH mud fluids of all three mud volcanoes. In a few high-pH samples, concentrations of up to ~5 mM are reached, which correspond to ~10% of seawater values (~54 mM). Drilling fluid (seawater) contamination is a likely source of these elevated Mg^2+^ values (Figure 2B) according to drilling fluid intrusion estimates based on perfluorocarbon tracer compounds (Fryer et al., 2017; Lever et al., in prep.). Mg^2+^ concentrations in high-pH fluids increase steeply near the seafloor, in the same intervals where pH values decrease, indicating significant fluid exchange, e.g., by diffusive mixing with seawater, in the top meters of sediment. By contrast, moderate-pH mud fluids show a gradual decrease but sustain significantly higher Mg^2+^ concentrations throughout the cored intervals. These Mg^2+^ concentrations cannot be explained with measured drilling fluid contamination (Fryer et al., 2017; Lever et al., in prep.) and indicate that moderate-pH pore fluids are a mixture of seawater and/or shallower sedimentary pore fluids and deeply-sourced serpentinitic fluids.

Sulfate concentrations in high-pH fluids (Figure 2C) show similar patterns to pH and magnesium, and in relation to chloride (Supplementary Figure S1B), i.e., changes toward seawater values (~28 mM) in the upper meters, and steady profiles below. Chloride concentrations below those of seawater in high-pH fluids, moreover, indicate fluid freshening due to dewatering of deep clay-bearing minerals (Supplementary Figure S1A). There is no clear trend in sulfate concentrations with distance to trench, as is evident from the fact that high-pH fluids from Fantangisña are depleted in sulfate (~4 mM), whereas those of Yinazao and Asùt Tesoru are only slightly lower (~27 mM) or even higher (~31 mM) than seawater values (28 mM; Mottl et al., 2003). By contrast, sulfate concentrations in moderate-pH fluids at Fantangisña show a striking variability, also in relation to chloride (Supplementary Figure S1), with values in holes U1497A and B decreasing steeply with sediment depth, while values from U1498A stabilize at ~25 mM, which is ~5-fold higher than in high-pH fluids of the same mud volcano. Sulfate concentration profiles on the flanks of Asùt Tesoru also vary between boreholes. Steep depth-related decreases to ~14 mM occur in the top 12 mbsf at U1495A and B, while more moderate decreases to ~15 mM and ~22 mM at ~30 mbsf occur at U1493B and U1494A, respectively. Unlike at Fantangisña, sulfate concentrations at Asùt Tesoru are higher in high-pH than in moderate-pH fluids.

DIC concentrations in high-pH fluids at Yinazao and Fantangisña remain mostly within a narrow range of 0 to 1 mM and are lower than those in seawater (Mottl et al., 2003; Figure 2D). By comparison, despite showing considerable scatter, measured DIC concentrations of high-pH fluids at Asùt Tesoru are higher (mostly 2–4 mM), in a range that is similar to or above seawater values. While DIC concentrations at shallow sediment depths and moderate-pH fluids at Fantangisña were in the same range as high-pH subsurface fluids, moderate-pH fluids at Asùt Tesoru consistently had 5–10-fold lower DIC concentrations than high-pH fluids.

DOC concentrations in high-pH fluids show straight subsurface profiles, with an increase in average concentration with distance to trench (Yinazao: 211 ± 51 μM; Fantangisña: 419 ± 79 μM; Yinazao: 1,684 ± 234 μM; Figure 2E). In moderate-pH fluids of Fantangisña and Asùt Tesoru, subsurface DOC concentrations also show no clear depth-related trends and have similar (Fantangisña; 317 ± 72 μM) or lower (Asùt Tesoru: 350 ± 195 μM) concentrations than in respective high-pH fluids. At Fantangisña, where measurements were also made in shallow sediment layers, there is a clear decrease in DOC concentrations toward the seafloor.

TOC contents in serpentinite muds are generally low, mostly scattering around ~0.01% dry weight, and do not show systematic differences between high- and moderate-pH fluids or between mud volcanoes (Figure 2F). Nonetheless, there are several outliers, including values with 2.1% (U1492C 1H2; Yinazao) and 3.3% dry weight (U1496B 8XCC; Asùt Tesoru). Comparing porosity-corrected DOC to TOC contents in subsurface fluids, there are clear differences between and within mud volcanoes. DOC accounts for similar contributions of TOC in high-pH subsurface fluids at Yinazao (1.5 ± 0.7%) and at high-pH (~2.0%) and moderate-pH (1.6 ± 1.3%) fluids of Fantangisña. By contrast, at Asùt Tesoru, the average DOC contribution to TOC is higher, i.e., 3.0 ± 1.2% in moderate-pH, and 13.1 ± 8.0% in high-pH subsurface fluids.

Cell counts reach values of ~10^6^ cells cm^−3^ in the upper tens of meters, but are mostly below or within an order of magnitude above the quantification limit of 20 cells cm^−3^ in deeper layers, both in high-pH and moderate-pH muds (Figure 2G).

### Concentration Profiles of Microbial Energy Substrates

Concentration profiles of potential microbial energy substrates show patterns with respect to depth below the seafloor and between high-pH and moderate-pH fluids that resemble those observed for pH, DIC, sulfate, and DOC.

Formate concentrations in high-pH fluids show straight subsurface profiles and increase with distance to trench (Yinazao: 1.8 ± 1.2 μM, Fantangisña: 7.0 ± 2.7 μM; Asùt Tesoru: 104 ± 11 μM; Figure 3A). Moderate-pH fluids from subsurface layers of Fantangisña are in a similar range (8.0 ± 3.7 μM) to high-pH fluids, and show the same characteristic decrease toward the seafloor that is also present in DOC concentrations. At Asùt Tesoru, however, moderate-pH, subsurface fluids have ~40-fold lower concentrations (2.7 ± 1.7 μM) than high-pH fluids.

Acetate concentrations (Figure 3B) show similar trends to formate, except that the characteristic decrease toward the seafloor is absent from shallow sediments of Fantangisña, Moreover, the increase in high-pH fluids with distance to trench is not as strong as for formate. Average acetate concentrations range between 1 and 10 μM in high-pH (Yinazao: 2.4 ± 1.6 μM; Fantangisña: 6.2 ± 1.6 μM) and moderate-pH fluids (Fantangisña: 4.9 ± 4.0 μM; Asùt Tesoru: 5.6 ± 4.4 μM), except in high-pH subsurface fluids of Asùt Tesoru, where acetate concentrations are clearly elevated (42.0 ± 4.7 μM).

Propionate concentrations (Figure 3C) show no clear trends related to depth below the seafloor, fluid pH, or distance to trench, and range mostly from 1 to 10 μM both in high-pH (Yinazao: 6.3 ± 1.7 μM; Fantangisña: 5.9 ± 1.2 μM; Asùt Tesoru: 4.4 ± 2.8 μM) and in moderate-pH fluids (Fantangisña: 5.8 ± 2.5 μM; Asùt Tesoru: 3.5 ± 4.0 μM). Propionate concentrations are relatively uniform across boreholes at Fantangisña, but vary significantly in moderate-pH fluids of Asùt Tesoru, where U1493B and U1491A have 5–10 times lower concentrations than U1495A and B. Similarly, there is an offset among high-pH fluids of Asùt Tesoru (U1496), where hole B has ~3 times lower concentrations than hole A.

Butyrate concentrations scatter in the submicromolar to low micromolar range (Figure 3D), showing no systematic relationship with depth below the seafloor, pH, or distance to trench. Average values of high-pH fluids are 1.5 ± 0.5 μM, 0.5 ± 0.2 μM, and 2.1 ± 0.3 μM at Yinazao, Fantangisña, and Asùt Tesoru, respectively. Average values of moderate-pH fluids are 0.6 ± 0.3 μM and 3.4 ± 3.6 μM at Fantangisña and Asùt Tesoru, respectively. The only notable trend is a linear decrease to the seafloor within the upper 10 mbsf at Fantangisña.

Other SCOAs were also detected in numerous samples (Supplementary Figure S2). Pyruvate was mainly detected in high-pH fluids of Asùt Tesoru (0.2 ± 0.1 μM). Valerate was detected in most high-pH fluid samples from Yinazao and Asùt Tesoru, with roughly 10-fold higher average concentrations in Asùt Tesoru (0.4 ± 0.2 μM vs. 0.04 ± 0.03 μM at Yinazao). Low (sub)micromolar concentrations of lactate were also present in most samples. Notably, lactate was the only SCOA with higher average concentrations in moderate-pH (1.5 ± 1.5 μM) than in high-pH fluids (0.7 ± 1.2 μM) of Asùt Tesoru.

Similar to formate, CH_4_ concentrations (Figure 3E) in high-pH fluids show relatively straight downcore profiles and increase dramatically with distance to trench (Yinazao: 2.4 ± 1.4 μM, Fantangisña: 10.4 ± 6.3 μM; Asùt Tesoru: 2,170 ± 1,630 μM). CH_4_ concentrations in moderate-pH subsurface fluids of Fantangisña and Asùt Tesoru are 10 to 100 times lower than in high-pH fluids from the same depths.

Similar to formate and CH_4_, H_2_ concentrations (Figure 3F) in high-pH fluids show a similar, strong increase with distance to trench (Yinazao: 16 ± 40 μM, Fantangisña: 380 ± 250 μM, Asùt Tesoru: 1,660 ± 1,560 μM). Compared to corresponding depths at Fantangisña and Asùt Tesoru, these H_2_ concentrations are approximately two orders of magnitude higher than those in moderate-pH fluids (Fantangisña: 22.2 ± 32.4 μM; Asùt Tesoru: 225 ± 660 μM).

Carbon monoxide (CO) concentrations were barely detectable in high-pH fluids of Yinazao, but were clearly above detection in high-pH fluids of the other two mud volcanoes (Fantangisña: 11 ± 3 μM; Asùt Tesoru: 7 ± 2 μM; Supplementary Figure S3). Compared to high-pH fluids, CO concentrations in moderate-pH fluids of Fantangisña and Asùt Tesoru are in a similar range or slightly higher (Fantangisña: 13 ± 9 μM; Asùt Tesoru: 12 ± 3 μM).

### Contribution of SCOAs to DOC

SCOAs make up ^~^10–30% of the C fraction of total DOC (Figure 4). The contribution of different SCOA species to the DOC pool varies with depth and pH. At Yinazao, the average SCOA fraction in high-pH fluids increases from ~15% at the seafloor to ~30% in the deepest samples. Propionate dominates the contribution in high-pH fluids (60–95% of SCOA C-pool), followed by acetate, butyrate, and then formate. Moderate pH-fluids at Fantangisña have trends similar to high-pH fluids at Yinazao, i.e., similar C contributions of SCOAs and propionate clearly dominating followed by acetate, but here the formate fraction is higher than the butyrate fraction. By comparison, SCOAs in high-pH fluids at Fantangisña account for a lower fraction of DOC, mainly due to a much lower C contribution of propionate, which barely exceeds acetate. The difference between moderate- and high-pH fluids is biggest at Asùt Tesoru. While moderate-pH fluid compositions fluctuate, and are variably dominated by acetate, propionate, and butyrate, high-pH fluids at Asùt Tesoru are consistently dominated by formate and acetate, which together account for 69–92% of the total SCOA-C.

### Microbial Activity Within Samples Based on Incubation Experiments

To check for measurable microbial activity, we incubated high-pH muds of all three mud volcanoes at *in situ* pH in the laboratory using formate as an energy substrate and monitored concentrations of the metabolites formate, H_2_, CH_4_, and DIC for 6 weeks. None of the samples showed significant changes in metabolite concentrations over time or between formate incubations (100 μM ^13^C-formate), killed controls (100 μM ^13^C-formate + sodium azide), and negative controls (no formate).

### SCOA Concentrations in High-PH Fluids in Relation to Distance to Trench and Slab Temperature

The very low cell numbers, the nearly constant concentration profiles of SCOAs in high-pH fluids, and the absence of measurable formate turnover in incubations raise the possibility that microbial activity is absent from high-pH subsurface muds. If so, then this raises the possibility that SCOAs measured in high-pH muds were thermogenically released from organic matter in the subducting slab and its sediments and/or produced by abiotic synthesis reactions linked to serpentinization of mantle rock of the Philippine Plate, and subsequently preserved over thousands of years during ascent. Under such a scenario, where all SCOAs in high-pH muds would be thermogenic or abiotic in origin, the changes in SCOA concentrations across mud volcanoes would reflect different environmental conditions deep within the subduction zone.

Our data are consistent with the possibility of SCOAs in high-pH fluids having deep origins. Cross-plots, in which previously measured formate and acetate concentrations from Conical Seamount (Haggerty and Fisher, 1992), a mud volcano that is located further away (86 km) from the Mariana Trench than Asùt Tesoru, are included, show that measured concentrations of formate and acetate in high-pH subsurface fluids are strongly correlated (power relationship; Figure 5B). Such a relationship is absent from moderate-pH samples (Figure 5A), or for propionate or butyrate in high-pH samples (*not shown*).

Next, we examined potential drivers behind the observed concentration trends in SCOAs in high-pH fluids across different mud volcanoes. Specifically, we investigated the relationship of formate, acetate, and propionate concentrations and mud volcano distance to the Mariana Trench, as a proxy for time since the initial subduction. Furthermore, we investigated the relationship between formate, acetate, and propionate concentrations and temperature in the subducting slab. The concentrations of formate and acetate, but not propionate, show a highly significant power relationship with distance to trench (Figure 6, upper panel) and with modeled *in situ* temperature in the underlying subducting slab (Figure 6, lower panel). Similarly, the ratios of formate to acetate, show strong power relationships with distance to trench and slab temperature (Supplementary Figure S4).

### Stable Isotopic Compositions of Bulk Carbon Pools

As with most other analytes, the subsurface δ^13^C-isotopic values of TOC, DOC, or DIC show no clear depth-related trends (Supplementary Figure S5), but instead indicate clear differences related to mud volcanoes and fluid pH (Figure 7).

Subsurface δ^13^C-TOC values fall into a narrow range (Yinazao, high-pH: −27.5 ± 1.9‰; Fantangisña: moderate-pH: −27.8 ± 1.5‰; high-pH: −27.6 ± 3.0‰; Asùt Tesoru: moderate-pH: −28.7 ± 0.6‰; high-pH: −26.9 ± 2.0‰), and thus do not differ significantly between mud volcanoes or between pH-muds within mud volcanoes (Mann Whitney Test; *p* > 0.05).

All subsurface δ^13^C-DOC data also fall within a narrow range, with the exception of high-pH fluids from Asùt Tesoru, where the average δ^13^C-DOC is 11.1–12.1‰ higher than in all other mud volcano fluids (Yinazao, high-pH: −25.2 ± 1.2‰; Fantangisña: moderate-pH: −25.4 ± 0.4‰; high-pH: −26.2 ± 1.7‰; Asùt Tesoru: moderate-pH: −25.5 ± 2.3‰; high-pH: −14.1 ± 1.6‰). This difference in δ^13^C-DOC between Asùt Tesoru high-pH fluids and all other mud volcano fluids is highly significant (*p* < 0.01).

Despite considerable scatter, subsurface δ^13^C-DIC data show a clear division between Asùt Tesoru and the other two mud volcanoes. At Asùt Tesoru, moderate-pH and high-pH fluids have highly similar δ^13^C-DIC ranges (moderate pH: −7.4 ± 3.6‰; high-pH: −7.6 ± 2.6‰). These values are significantly higher than in high-pH fluids of Yinazao (−16.3 ± 1.9‰) and Fantangisña (−15.3 ± 1.7‰; note: no data from moderate-pH fluids), which did not differ significantly from each other.

Comparing subsurface δ^13^C-isotope data to each other reveals several trends. Overall, the mean δ^13^C-DOC is consistently higher than the mean δ^13^C-TOC. This difference is small (1.5–2.3‰) in muds of Yinazao and Fantangisña, and moderate-pH muds of Asùt Tesoru, but comparatively large (12.8‰) in high-pH muds of Asùt Tesoru (−14.1 ± 1.6‰ vs. −26.9 ± 2.0‰). Comparing δ^13^C-DIC to δ^13^C-TOC and δ^13^C-DOC within each location and pH category shows that the δ^13^C-DIC is consistently higher than the δ^13^C-TOC and δ^13^C -DOC. The average difference between δ^13^C-DIC and δ^13^C-TOC is lower in high-pH muds of Yinazao (−11.2‰) and Fantangisña (−12.3‰) compared to moderate-pH (−20.5‰) and high-pH muds (−19.3‰) at Asùt Tesoru. The average differences between δ^13^C-DIC and δ^13^C-DOC are in a similar range in high-pH muds of Yinazao (8.9‰) and Fantangisña (10.9‰) and in moderate-pH muds of Asùt Tesoru (18.1‰). The only exception is again high-pH mud of Asùt Tesoru, where the average δ^13^C-DIC is only 6.6‰ higher than the average δ^13^C-DOC.

## Discussion

Concentrations of potential microbial energy sources, such as formate, acetate, CH_4_, H_2_, and DOC, in deeply sourced high-pH fluids of Mariana forearc serpentinite mud volcanoes increase systematically with distance to the Mariana Trench and with underlying slab temperature. Yet, despite these increases, there are no clear indications of metabolically active microbial populations. Downward SCOA concentrations in high-pH subsurface muds of all three mud volcanoes show no clear changes, and neither do concentrations of other potential metabolites, such as sulfate, DIC, and DOC. Though an interpretation of the dissolved CH_4_ and H_2_ data is confounded by potential outgassing during core retrieval, these gases also show no clear depth-related production or consumption profiles. Microbial populations, quantified by microscopic counting, are mainly around the minimum quantification limit of 20 cells cm^−3^. Local cell population peaks in subsurface high-pH fluids have to be interpreted with caution, given the local detection of millimolar concentrations of seawater-derived Mg^2+^, and evidence of significant contamination of sediment porewater by drilling fluid (surface seawater) containing cell concentrations of 10^5^-10^6^ cells cm^−3^ (Lever et al., *in prep*.). Yet, even if the local peaks in cell counts accurately reflect *in situ* cell populations, these cells may not be metabolically active due to the highly alkaline *in situ* pH.

If significant microbial activity is absent, then this would mean that concentrations and compositions of potential microbial electron donors, such as SCOAs, CH_4_, H_2_, and CO, and electron acceptors, such as sulfate and DIC, in high-pH fluids could provide useful insights into deep, non-biological processes in the subducting slab and the overlying forearc mantle. For these high-pH fluids to be indeed informative, non-biological alterations of electron donor and acceptor compositions during the tens of thousands of years of mud fluid ascent would need to be absent or sufficiently slow to not overprint original trends. In the following sections we first investigate possible explanations for the absence of detectable microbial activity in this subseafloor environment, focusing on free energy yields of catabolic reactions and the environmental variables pressure, temperature, pH, and fluid mixing during ascent. Afterward we discuss possible deep sources and production mechanisms of measured SCOAs, as well as sulfate, methane, and bulk carbon pools, across the Mariana forearc.

### Free Energies of Catabolic Reactions

To determine whether catabolic reactions are thermodynamically favorable, we calculated Gibbs energies for a range of reactions at *in situ* temperature, pressure, and pH in cores, as well as at estimated temperature, and pressure in the subducting slab using the *in situ* pH in the cores (Table 2). We include three respiration reaction types (methanogenesis, sulfate reduction, acetogenesis) that are important in marine serpentinitic systems (Brazelton et al., 2006; Quéméneur et al., 2014; Rempfert et al., 2017; Ijiri et al., 2018; Lang et al., 2018). Besides SCOAs, we include H_2_, CO, and methane as substrates of microbial respiration, because of the ubiquitously high concentrations of H_2_ and CH_4_ and the detectable concentrations of CO in some of the high-pH muds. We also calculate free energy yields for the fermentative breakdown of propionate and butyrate, and the fermentative conversion of glucose to propionate and butyrate, as fermentation reactions are also likely to occur in serpentinitic environments (e.g., Kohl et al., 2016; Brazelton et al., 2017; Rempfert et al., 2017). While the currently known temperature limit of microbial life is 122°C (Takai et al., 2008), and thus below the slab temperatures at Fantangisña and Asùt Tesoru, we nonetheless include calculations at all slab temperatures. This is because it cannot be ruled out that deep life exists at >122°C. Furthermore, many of the reactions in Table 2 can also operate thermochemically or abiotically at high temperature and thus provide insights into potential non-biological sources and production pathways discussed later.

**Table 2:** Mean Gibbs energies (ΔG_*r*_*)* (±standard deviation) of potential catabolic reactions in high-pH fluids of each mud volcano.

***e^**−**^* donors by reaction type**	**Reaction**	**Yinazao**		**Fantangisña**		**Asùt Tesoru**	
		**2.6°C**	**80°C**	**3.5°C**	**150°C**	**2.2°C**	**250°C**
Sulfate reduction							
H_2_	4 H_2_ + SO${}_{4}^{2-}$+ H^+^ → 4 H_2_O + HS^−^	−95 ± 16	−57 ± 20	−127 ± 10	−77 ± 15	−140 ± 6	−63 ± 12
Formate	4 HCOO^−^+ SO${}_{4}^{2-}$ → HS^−^+ 4 CO${}_{3}^{2-}$+ 3 H^+^	−111 ± 10	−149 ± 9	−129 ± 5	−165 ± 5	−163 ± 3	−253 ± 5
Acetate	CH_3_COO^−^+ SO${}_{4}^{2-}$ → HS^−^+ 2 CO${}_{3}^{2-}$+ 2 H^+^	−69 ± 3	−114 ± 4	−73 ± 2	−134 ± 1	−87 ± 1	−207 ± 3
Propionate	2 CH_3_CH_2_COO^−^+ 3 SO${}_{4}^{2-}$+ 2 H_2_O → 3 HS^−^+ 6 CO${}_{3}^{2-}$+ 2 H_2_ + 7 H^+^	−230 ± 12	−379 ± 15	+225 ± 10	−449 ± 11	−252 ± 4	−677 ± 9
Butyrate	2 CH_3_CH_2_CH_2_COO^−^+ 4 SO${}_{4}^{2-}$+ 4 H_2_O → 4 HS^−^+ 8 CO${}_{3}^{2-}$+ 4 H_2_ + 10 H^+^	−310 ± 20	−533 ± 26	−277 ± 16	−604 ± 17	−322 ± 7	−947 ± 14
Carbon monoxide	4 CO + SO${}_{4}^{2-}$+ 4 H_2_O → 4 CO${}_{3}^{2-}$+ HS^−^+ 7 H^+^	−322 ± 5	−397 ± 7	−370 ± 7	−444 ± 4	−399 ± 5	−552 ± 11
Methane	CH_4_ + SO${}_{4}^{2-}$ → HS^−^+ CO${}_{3}^{2-}$+ H^+^ + H_2_O	−27 ± 4	−43 ± 6	−32 ± 2	−58 ± 2	−52 ± 2	−117 ± 3
Methanogenesis							
H_2_	4 H_2_ + CO${}_{3}^{2-}$+ 2 H^+^ → CH_4_ + 3 H_2_O	−68 ± 14	−10 ± 18	−95 ± 10	−15 ± 15	−88 ± 5	+59 ± 9
Formate	4 HCOO^−^+ H_2_O → CH_4_ + 3 CO${}_{3}^{2-}$+ 2 H^+^	−84 ± 8	−102 ± 11	−98 ± 4	−103 ± 5	−112 ± 2	−130 ± 4
Acetate	CH_3_COO^−^+ H_2_O → CH_4_ + CO${}_{3}^{2-}$+ H^+^	−42 ± 4	−66 ± 5	−41 ± 2	−72 ± 2	−36 ± 1	−84 ± 2
Carbon monoxide	4 CO + 5 H_2_O → CH_4_ + 3 CO${}_{3}^{2-}$+ 6 H^+^	−295 ± 5	−350 ± 6	−339 ± 6	−382 ± 5	−346 ± 6	−430 ± 9
Acetogenesis							
H_2_	4 H_2_ + 2 CO${}_{3}^{2-}$+ 3 H^+^ → CH_3_COO^−^+ 4 H_2_O	−25 ± 16	+57 ± 20	−53 ± 11	+59 ± 16	−52 ± 6	+145 ± 11
Formate	4 HCOO^−^ → CH_3_COO^−^+ 2 CO${}_{3}^{2-}$+ H^+^	−42 ± 6	−35 ± 8	−56 ± 3	−29 ± 5	−76 ± 1	−45 ± 3
Formate + H_2_	2 HCOO^−^+ 2 H_2_ + H^+^ → CH_3_COO^−^+ 2 H_2_O	−33 ± 8	+12 ± 10	−55 ± 6	+15 ± 10	−64 ± 3	+50 ± 6
Carbon monoxide	4 CO + 4 H_2_O → CH_3_COO^−^+ 2 CO${}_{3}^{2-}$+ 5 H^+^	−252 ± 4	−283 ± 5	−296 ± 5	−309 ± 4	−311 ± 4	−344 ± 9
Carbon monoxide + H_2_	2 CO + 2 H_2_ → CH_3_COO^−^+ H^+^	−135 ± 8	−92 ± 10	−172 ± 5	−104 ± 8	−181 ± 4	−74 ± 8
Fermentation							
Propionate	CH_3_CH_2_COO^−^+ 3 H_2_O → CH_3_COO^−^+ CO${}_{3}^{2-}$+ 3 H_2_ + 2 H^+^	+2 ± 12	−59 ± 16	+25 ± 8	−64 ± 12	+32 ± 4	−116 ± 9
Butyrate	CH_3_CH_2_CH_2_COO^−^+ 2 H_2_O → 2 CH_3_COO^−^+ 2 H_2_ + H^+^	−16 ± 3	−60 ± 11	+7 ± 5	−55 ± 7	−14 ± 3	−89 ± 6
Glucose to propionate	C_6_H_12_O_6_ + 5 H_2_O → CH_3_CH_2_COO^−^+ 3 CO${}_{3}^{2-}$+ 5 H_2_ + 7 H^+^	−358 ± 21	−519 ± 26	−323 ± 15	−554 ± 20	−340 ± 7	−765 ± 14
Glucose to butyrate	C_6_H_12_O_6_ + 2 H_2_O → CH_3_CH_2_CH_2_COO^−^+ 2 CO${}_{3}^{2-}$+ 2 H_2_ + 5 H^+^	−365 ± 9	−467 ± 11	−361 ± 8	−512 ± 10	−375 ± 3	−659 ± 6
Carbon monoxide production							
H_2_	H_2_ + CO${}_{3}^{2-}$+ 2 H^+^ → CO + 2 H_2_O	+57 ± 4	+85 ± 5	+61 ± 3	+92 ± 4	+65 ± 1	+122 ± 3
Formate	HCOO^−^+ H^+^ → CO + H_2_O	+53 ± 2	+62 ± 2	+60 ± 1	+70 ± 1	+59 ± 1	+75 ± 2
Formate oxidation							
Formate	HCOO^−^+ H_2_O → CO${}_{3}^{2-}$+ H_2_ + H^+^	−4 ± 5	−23 ± 6	−1 ± 2	−22 ± 3	−6 ± 1	−47 ± 3

*Calculations were done at in situ temperature, pressure, and pH of cores during sampling (left column) and under slab conditions (right column). Estimated pH values under slab conditions were 11.2 for Yinazao, 11.0 for Fantangisña, and 12.5 for Asùt Tesoru. In situ pressures were calculated based on hydrostatic pressure assuming a seawater density of 1.013 g cm^−3^ and a mud density of 1.8 g cm^−3^ (based on Fryer et al., 2018b). Thermodynamically unfavorable reactions with positive Gibbs energies are shaded*.

Our calculations indicate that the majority of reactions are thermodynamically favorable in all three mud volcanoes under both core and slab conditions (Table 2). Gibbs energies are mostly more negative than the theoretical minimum amount of energy that can be conserved per biochemical reaction (−20 to −10 kJ mol^−1^; Schink and Thauer, 1988; Hoehler et al., 2001), also known as the “biological energy quantum” (BEQ; Thauer and Morris, 1984). Sulfate reduction is exergonic from all substrates, with reactions from SCOAs and CO in many cases being highly exergonic (ΔG_*r*_ < −100 kJ mol^−1^), especially at slab temperatures. Methanogenesis from formate, acetate, and CO is also always exergonic. By contrast, methanogenesis from H_2_ (+${\text{CO}}_{3}^{2-}$) is thermodynamically favorable under core conditions but in the range of the BEQ or even endergonic under slab conditions. Acetogenesis from formate, CO, and CO+H_2_ is also always exergonic. Acetogenesis with H_2_ (+${\text{CO}}_{3}^{2-}$) or formate+H_2_ as substrates is furthermore exergonic at core conditions, whereas the reverse reaction, acetate oxidation to H_2_ (+${\text{CO}}_{3}^{2-}$) or formate+H_2_, is thermodynamically favorable under slab conditions. Fermentation of glucose to propionate or butyrate is highly favorable under all conditions, whereas propionate or butyrate fermentations to acetate are only clearly exergonic under slab conditions. The only reactions that are always endergonic, whether under core or slab conditions, are reactions involving the conversion of H_2_+${\text{CO}}_{3}^{2-}$ or formate to CO (ΔG_*r*_ > +50 kJ mol^−1^). On the other hand, the reverse reaction is clearly exergonic, underscoring the potential for CO oxidation as a catabolic pathway in serpentinitic fluids (Morrill et al., 2014). Finally, the oxidation of formate to ${\text{CO}}_{3}^{2-}+$H_2_ is close to thermodynamic equilibrium under core conditions, but exergonic under slab conditions.

In summary, given that many known catabolic reactions have significant free energy yields under core and slab conditions, the very low to absent microbial activity in high-pH fluids of the three mud volcanoes cannot be explained with absence of suitable substrates for energy-yielding catabolic reactions.

### Potential Physiological Limits to Microbial Life in Subseafloor Serpentinites

Next we will discuss the potential roles of pressure, temperature, pH, and lack of fluid mixing as variables that limit the proliferation of microbial life in serpentinite mud volcanoes of the Mariana Forearc.

Among these variables, pressure alone is perhaps the least likely to be a strong limiting factor. We calculate *in situ* pressures of 2,740, 2,670–2,820, and 3,300–3,500 bar for the subducting slabs of Yinazao, Fantangisña, and Asùt Tesoru, respectively. While these values are higher than those in surface sediments of the deepest part of the world's oceans, the Mariana's Trench, or any subseafloor cores that have been recovered to date, laboratory experiments suggest that microbial life can remain metabolically active at >10,000 bar (Sharma et al., 2002) and survive even higher pressures (20,000–30,000 bar; Hazael et al., 2016). Furthermore, experiments suggest that the ability to survive high pressure (>10,000 bar) can evolve rapidly, even in microbial strains that are not pre-adapted to elevated pressure, such as *Escherichia coli* or *Shewanella oneidensis* (Vanlint et al., 2011; Hazael et al., 2014).

Temperature is a more likely limiting factor. As aforementioned, the slab temperatures at Fantangisña (150°C) and Asùt Tesoru (250°C) are above the known temperature limit of life (122°C; Takai et al., 2008). Furthermore, even though temperature in the subducting slab at Yinazao (80°C) is below this temperature limit, there is evidence that microbial life ceases at or below 80°C in energy-limited subsurface environments (Head et al., 2003; Inagaki et al., 2015; Møller et al., 2018; Heuer et al., 2019), despite heat-driven increases in the release of energy substrates from thermogenic (e.g., Wellsbury et al., 1997; Parkes et al., 2007) or abiotic reactions (e.g., McCollom and Seewald, 2001, 2003a). This lower temperature limit in the subsurface may exist for the following reasons. Rates of biomolecule-damage increase exponentially with temperature (e.g., Lindahl and Nyberg, 1972; Wolfenden et al., 1998; Steen et al., 2013). Cells may adjust their biomolecule compositions toward building blocks with higher thermal stability, e.g., DNA with higher GC content, and amino acids with lower racemization rates at elevated temperature. Yet, the resulting enhanced thermal stability is minor compared to the impact of temperature on biomolecule damage rates. For instance, over a temperature increase from 2 to 80°C, increases in racemization rates may vary by one order of magnitude between amino acids; yet this difference is small considering that racemization rates of all amino acids increase by ~3–4 orders of magnitude over this temperature interval (Steen et al., 2013; Lever et al., 2015). Due to this dramatic increase in energy needed for biomolecule repair, it has been proposed that the upper temperature limit of microorganisms in energy-limited subsurface habitats is lower than in energy-replete environments, e.g., laboratory growth media or hydrothermal vent chimneys (Lever et al., 2015). Yet, crucially, the temperature argument only applies if the mud fluids remain free of microbial recolonization during ascent. If there is any significant microbial re-inoculation of cooled but presumably sterile serpentinite mud fluids from sediments or other fluids during ascent, then these microbial colonizers could potentially thrive as a result of the high concentrations of energy substrates and the high free energy yields of catabolic reactions. Indeed, geochemical data from our study, and from a study on a borehole observatory at the nearby South Chamorro Seamount (IODP Site 1200C; Kawagucci et al., 2018), suggest at most minimal recolonization of Mariana forearc serpentinite muds during ascent from the subducting slab.

The other potentially important factor is pH. Porewater pH values in high-pH fluids (Yinazao: ~10.7; Fantangisña: ~11.2; Asùt Tesoru: ~12.4) and for the underlying subducting slab (Yinazao: ~11.2; Fantangisña: ~11.0; Asùt Tesoru: ~12.5; Fryer et al., 2018c) are in the growth range of microbial pure cultures (Takai et al., 2005; Suzuki et al., 2014; current record: pH 12.5, Takai et al., 2001; reviewed in Takai, 2019). Furthermore, there have been several enrichments of microorganisms and microbial DNA detections based on natural serpentinitic fluids with a pH≥12 (e.g., Crespo-Medina et al., 2014; Morrill et al., 2014; Kohl et al., 2016; Brazelton et al., 2017; Suzuki et al., 2017) including mud and borehole fluids of the nearby South Chamorro Seamount (Curtis et al., 2013; Kawagucci et al., 2018). Based on measured pH values, microbial life should therefore be possible in high-pH muds of the three mud volcanoes. Nonetheless, it is possible that the *in situ* pH is considerably higher than measured. The *in situ* pH in mud fluids at South Chamorro, which has the same measured pH value as Asùt Tesoru (12.5), has been estimated to be significantly higher (13.1; Mottl, 2009), and thus significantly above the known pH limit of microbial life. Furthermore, it is possible that high pH poses an effective barrier to microbial recolonization from sediment or other fluids during ascent, if colonizing cells are not adapted to such high pH.

In conclusion, elevated temperature (80–250°C) and extreme pH can explain the (near) absence of microbial life in ascending serpentinite mud fluids of the Yinazao, Fantangisña, and Asùt Tesoru mud volcanoes. If temperature functions as a natural sterilizing agent in the subducting slab, then this would indicate minimal recolonization of fluids during their long ascent (Yinazao: ~50,000 years, Asùt Tesoru: ~130,000 years), during which their temperatures drop within the known limits of life (Fryer et al., 2018b; Table 2). The absence of recolonization could be due to recruitment limitation, e.g., no or only minimal mixing with other fluids or sediments containing microbial life. This notion is supported by the very low cell numbers and absence of clear microbial activity in moderate-pH fluids of the same three mud volcanoes. Alternatively, microbial life present in other fluids or sediments that come into contact with high-pH serpentinite fluids during ascent may not tolerate the high pH of serpentinite fluids.

### Potential Sources and Production Mechanisms of Deep SCOAs

We document a strong increase in total SCOA concentrations and systematic shift in the composition of SCOAs in high-pH serpentinite muds that is correlated with distance to the Mariana Trench and slab temperature (Figures 3–6). The shift in SCOA compositions is mainly due to concentration increases of acetate and especially formate (Figure 5). Concentrations of propionate, which is the dominant SCOA at Yinazao, butyrate, or other SCOAs do not change substantially with distance to trench or slab temperature. Lactate (and CO) concentrations are even overall higher in moderate-pH compared to high-pH fluids.

The factors that are responsible for the strong changes in SCOA compositions are unclear. Distance to trench *per se* is an unlikely driver, but distance to trench is correlated with time since initial subduction and with pressure. SCOA compositions could evolve as production rates of different SCOAs change as a function of time and pressure. However, time and pressure effects are unlikely to be the main drivers behind the observed trends. Relative to Yinazao, distance to trench only changes by ~13, ~31, and ~56% at Fantangisña, Asùt Tesoru, and Conical Seamount, respectively, and pressure also only increases by at most ~30% from Yinazao to Asùt Tesoru (no data for Conical). By contrast, formate and acetate concentrations increase exponentially by ~3 and ~2 orders of magnitude, respectively, from Yinazao to Conical Seamount (Figures 5, 6). This exponential increase is consistent with the well-known mathematical relationship between temperature and rates of chemical reactions (Arrhenius equation), and suggests that temperature is the main driver behind the observed differences in formate and acetate concentrations.

As slab temperatures increase, the sources of individual SCOAs and/or the balance between production and consumption of individual SCOAs appear to change. Concentrations of formate, acetate, butyrate, and CH_4_ in the low micromolar and submicromolar range in high-pH muds of Yinazao are in the typical range of non-methanogenic marine sediments (Glombitza et al., 2014, 2015), and provide no indications of significant additional, non-diagenetic inputs. Only propionate and H_2_ stand out, albeit only slightly. Propionate concentrations (~5–10 μM) exceed those in typical marine sediments (≤2 μM; Glombitza et al., 2019), and are consistent with a low-temperature thermogenic propionate input (80°C; Carothers and Kharaka, 1978). H_2_ concentrations (~1–100 μM) are clearly higher than in typical marine sediments (<1 μM; e.g., Hoehler et al., 1998; Lin et al., 2012) and are consistent with a low-temperature serpentinization fluid input (Mayhew et al., 2013; Miller et al., 2017). Going from Yinazao (80°C) to Fantangisña (150°C), the clear increases in formate (to ~10 μM) and especially H_2_ concentrations (to ~100–1,000 μM) are consistent with temperature-driven increases in serpentinization rates. By contrast, a significant stimulation of thermogenic processes, which would be expected to clearly increase concentrations of others SCOAs, is not evident.

A big shift in SCOA compositions occurs from Fantangisña (150°C) to Asùt Tesoru (250°C), where formate and acetate concentrations increase 15- and 7-fold, and methane and H_2_ concentrations increase ~200- and 4-fold, respectively (Figure 3). The observed increases in formate and acetate concentrations continue as temperatures increase further from Asùt Tesoru to Conical Seamount (350°C). For reasons, that we discuss in the next paragraphs, we believe that these strong increases in formate and acetate concentrations are driven by serpentinization.

High formate concentrations are a common feature of serpentinizing systems (Mottl et al., 2003; McCollom and Bach, 2009; Schrenk et al., 2013), and are attributed to a metastable equilibrium of formate with H_2_ and ${\text{CO}}_{3}^{2-}$ (McCollom and Seewald, 2001, 2003a). Hereby high formate concentrations result from the chemical reaction of H_2_, produced during reactions of iron(II)-rich minerals with water, with ${\text{CO}}_{3}^{2-}$ and H^+^. This reaction is equivalent to the reversal of biological formate oxidation, which is shown in Table 2. While earlier studies suggest that formate equilibration with H_2_+${\text{CO}}_{3}^{2-}+$H^+^ takes place at 175–300°C (McCollom and Seewald, 2001, 2003a), recent laboratory incubations of olivine-rich rocks indicate significant formate production from H_2_+CO_2_ at only 100°C (Miller et al., 2017). These data are in line with our calculations, which suggest that formate is at or near thermodynamic equilibrium with H_2_+${\text{CO}}_{3}^{2-}+$H^+^ in cores, but not under estimated slab temperature, pressure, and pH (Table 2). The fact that this is even true for Yinazao, suggests that equilibration between formate and H_2_+${\text{CO}}_{3}^{2-}+$H^+^ continues at temperatures significantly below 80°C.

The elevated acetate concentrations at Asùt Tesoru and Conical Seamount are more difficult to interpret than the elevated formate concentrations, as acetate is produced by serpentinization-related processes (Miller et al., 2017), and also is the dominant SCOA produced by thermogenic breakdown of OM (e.g., Kharaka et al., 1993; Shebl and Surdam, 1996; Dhillon et al., 2005). Though thermogenic acetate could be produced by thermal breakdown of OM from subducting sediment or mantle rock during serpentinitic alteration (Kelley and Früh-Green, 2001; McDermott et al., 2015), we consider abiotic synthesis processes to be the most likely source. This is because high rates of thermogenic acetate production are typically accompanied by significant increases in propionate and/or butyrate concentrations, which were not observed. Furthermore, isotopic data from Asùt Tesoru (Sissmann et al., *in preparation*) support an abiotic serpentinization-related production of acetate (and formate), as δ^13^C-values of both vary from ~0 to ~5‰, which is in the range of DIC, but considerably higher than the δ^13^C values of TOC and DOC (Figure 7).

### Geochemical Trends in Sulfate, Methane, and Bulk Organic Carbon Pools

Besides the increase in H_2_ and formate concentrations, a striking change between Yinazao and Fantangisña high-pH mud fluids is the 80% decrease in sulfate concentrations, from close to seawater values (25 mM) at Yinazao to <5 mM at Fantangisña. This decrease is consistent with anhydrite (CaSO_4_) precipitation, which has been proposed to remove most or all seawater sulfate from fluids during subduction in the Mariana forearc, and is only partially reversed by re-dissolution during fluid ascent (Kawagucci et al., 2018). By comparison, sulfate removal by thermochemical sulfate reduction, which is thermodynamically favorable in all mud volcanoes (Table 2), is an unlikely driver, both based on previous studies, which suggest that this process is inhibited in high-pH, serpentinitic systems (Seyfried et al., 2007), and given that sulfate concentrations increase again at higher temperatures. This strong increase in sulfate concentrations to values that exceed those in seawater in high-pH fluids of Asùt Tesoru (~31 mM; this study) and Conical Seamount (up to ~47 mM; Shipboard Scientific Party, 1990) is enigmatic. A possible source is redissolution of anhydrite during fluid ascent. Furthermore, antigorite, which forms at >200°C above the slab at Asùt Tesoru (Debret et al., 2019), and breaks down during serpentinization to generate oxidizing conditions that lead to sulfate production (Debret and Sverjensky, 2017), might explain the high sulfate concentrations in high-pH fluids of Asùt Tesoru and Conical Seamount. This same mechanism of antigorite breakdown could also explain the increase in DIC concentrations at Asùt Tesoru (Figure 2D) and the high alkalinities of 33–62 meq kg^−1^ in high-pH subsurface pore fluids of Conical Seamount (Shipboard Scientific Party, 1990).

As mentioned earlier, average CH_4_ concentrations in high-pH subsurface fluids increase ~200-fold from Fantangisña to Asùt Tesoru. Earlier data on high-pH fluids from Conical Seamount suggest that CH_4_ concentrations are within the range of Asùt Tesoru (Shipboard Scientific Party, 1990), which would indicate no substantial further increases in CH_4_ release as slab temperatures increase from 250 to 350°C. The sources of these very high CH_4_ concentrations are controversial, and could in principle be abiotic or thermogenic. Although our SCOA data suggest that thermogenic breakdown of OM is not a dominant process of C cycling in high-pH fluids of Asùt Tesoru or Conical Seamount, potential mechanisms of abiotic CH_4_ production are also unclear. Our thermodynamic calculations indicate that abiotic CH_4_ formation from H_2_ (+${\text{CO}}_{3}^{2-}$) under aqueous conditions in the slab is an endergonic process (Table 2). By contrast, production of CH_4_ by thermal decarboxylation of acetate, a reaction that has the same stoichiometry as biological methanogenesis from acetate (Kharaka et al., 1993) and is catalyzed by magnetite (McCollom and Seewald, 2003b), is thermodynamically favorable (Table 2). Yet, this reaction would require the production of millimolar acetate concentrations, and for >95% of this acetate to then be decarboxylated to CH_4_ + ${\text{CO}}_{3}^{2-}$. If recent field studies on multiple submarine locations and laboratory experiments with olivine are a good indication, then it is more likely that most of the CH_4_ in serpentinitic fluids of Asùt Tesoru and Conical Seamount is released from fluid inclusions within serpentinized mantle rock (McDermott et al., 2015; McCollom, 2016; Wang et al., 2018). In addition, CH_4_ could form in thermodynamically distinct chemical microenvironments with H_2_-rich vapors, e.g., serpentinization fronts (McCollom et al., 2016), rock fractures, or rock pores (Etiope and Whiticar, 2019). Akin to CH_4_ from fluid inclusions, abiotically produced CH_4_ from these microenvironments could then be liberated into mud fluids as a result of serpentinitic rock-alteration.

The TOC, DOC, and DIC isotopic compositions provide general insights into the sources of organic and inorganic carbon at Yinazao, Fantangisña, and Asùt Tesoru. Similar δ^13^C-values (range: −21 to −30‰, with most values between −26 to −30 ‰) and similar, for the most part very low (0.01 wt. %) TOC contents indicate similar origins of TOC across all three mud volcanoes, including high-pH and moderate-pH muds (Figure 7, Supplementary Figure S5). These values are mostly lower than TOC of marine phytoplankton and suggest that—if they are of sedimentary origin—there is a significant terrestrial TOC contribution. While δ^13^C-values of sedimentary TOC in the Mariana Trench are dominated by marine phytoplankton-derived organic carbon with higher isotopic values (−19 to −21‰), local layers with potentially significant terrestrial contributions (−24 to −25‰) were noted previously (Luo et al., 2017). Thus, it is possible that terrestrial TOC or certain ^13^C-enriched marine TOC fractions are selectively enriched during subduction, as a result of greater resistance to microbial and heat-driven degradation processes. Alternatively, given that the TOC contents and δ^13^C values are in the same range as those from other oceanic basement rocks [mostly 0–0.02 wt. %, −25 to −30‰; compiled in (Delacour et al., 2008)], it is also possible that most of the TOC is indigenous to the overlying plate. Heat-driven serpentinitic alteration would then release soluble fractions of mantle rock-bound TOC into solution and drive the increase in DOC concentrations in high-pH fluids from Yinazao to Asùt Tesoru. At Asùt Tesoru, the 4-fold increase in DOC concentrations compared to Fantangisña can, however, only in part be explained by release of rock-bound indigenous organic carbon. Here the strong isotopic offset (~+10‰) of DOC relative to TOC suggests that a major fraction of DOC derives from a source that is significantly heavier than TOC. The contributions of formate and acetate, which account for ~10–12% of the DOC and have ^13^C-isotopic values in the range of DIC at Asùt Tesoru, can only explain an isotopic offset of ~+2‰. Thus, the origin of a major component of the DOC pool in high-pH fluids of Asùt Tesoru remains unknown.

## Conclusions

Our study produces novel insights into the controls on the production of microbial energy substrates, in particular SCOAs, in deeply buried subducting slab environments. Despite the presence of high microbial energy substrate concentrations and significant Gibbs energy yields of a wide range of catabolic reactions, microorganisms are rare or absent, and no unequivocal evidence for microbial activity could be detected. This has implications for our understanding of deeply buried serpentinitic environments as potential habitats or even deep hotspots of microbial life, and suggests that the combination of temperature, highly alkaline pH, and dispersal limitation may strongly limit microbial population size in these environments. Instead, due to the absence of strong diagenetic alteration during fluid ascent over tens of millennia, high-pH fluids from mud volcanoes offer a unique window into the abiotic production mechanisms of microbial energy substrates by serpentinization and by serpentinization-related processes within subduction zones. Future studies will reveal the mechanisms by which some of these energy compounds, e.g., acetate, CH_4_, are produced, which unknown sources contribute to the strongly elevated DOC concentrations in high-temperature, high-pH fluids, and what the origins of the still enigmatic moderate-pH fluids are.

## Data Availability

The datasets generated for this study are available on request to the corresponding author.

## Author Contributions

PE and ML designed the study. PE, KT, OS, SSu, CM, SSa, PS, ET, and ML took samples, performed measurements, and/or contributed data analyses. SB, CG, BJ, and YM provided technical advice and support with the analyses. PE and ML wrote the manuscript with input from all co-authors.

### Conflict of Interest Statement

The authors declare that the research was conducted in the absence of any commercial or financial relationships that could be construed as a potential conflict of interest.

## Supplementary Material

Supplementary Figure S1 **(A)** Chloride concentration depth profiles at all three mud volcanoes. **(B)** Depth profiles of sulfate-to-chloride concentration ratios (both mM). High-pH fluids (summit sites) are indicated by red marker symbols, moderate-pH fluids (flank sites) by black marker symbols. The dashed gray vertical lines indicate typical seawater chloride concentrations (559 mM) and seawater sulfate-to-chloride ratios [0.0517; assumes [sulfate] = 28.9 mM]. The dashed red vertical lines indicate chloride concentration asymptotes for high-pH fluids [Yinazao: 460 mM; Fantangisña: 480 mM; Asùt Tesoru (U1496A): 540 mM]. Chloride concentrations in high-pH fluids that are significantly below those in seawater indicate a potential freshwater contribution from dewatering of deeply buried clay minerals.

Supplementary Figure S2 Concentration profiles of pyruvate, lactate, and valerate across boreholes of the three mud volcanoes.

Supplementary Figure S3 Concentration profiles of CO across boreholes of the three mud volcanoes.

Supplementary Figure S4 Concentration ratios of formate-to-acetate, formate-to-propionate, and acetate-to-propionate vs. temperature in the subducting slab (upper panel) and distance to the Mariana Trench (lower panel).

Supplementary Figure S5 δ^13^C-isotopic compositions of TOC, DOC, and DIC from moderate-pH (left column) and high-pH (right column) mud fluids of three serpentinite mud volcanoes drilled during IODP Expedition 366.
