# Supplementary material for: Origin of Short-Chain Organic Acids in Serpentinite Mud Volcanoes of the Mariana Convergent Margin
Source: Front Microbiol. 2019 Jul 26;10:1729. doi: 10.3389/fmicb.2019.01729 (PMC6677109; doi:10.3389/fmicb.2019.01729)
Supplement: Supplementary file 1 [file Table_1.docx]

Supplementary Table 1: Standards molal thermodynamic data of aqueous educts and products used in thermodynamic calculations.

| **Compound** | **∆G_f_° (kJ mol^-1^)** | **∆H_f_° (kJ mol^-1^)** | **∆V_f_° (cm^3^ mol^-1^)** | **Reference** |
| --- | --- | --- | --- | --- |
| Proton (H^+^) | 0.0 | 0.0 | 0.0 | Shock et al. (1997) |
| Hydrogen (H_2_) | -528.34 | -675.69 | -5.02 | Wagman et al. (1982)  Shock and Helgeson (1990) |
| Water | -237.2 | -285.8 | 18.0 | Amend and Shock (2001) |
| Carbonate (CO_3_^2-^) | -586.9 | -692.0 | 24.6 | Shock et al. (1997) |
| Carbon monoxide | -120.08 | -89.48 | 32.8 | Fenn (1971),  Oelkers et al. (1995) |
| Formate | -351.0 | -425.7 | 26.2 | Shock and Helgeson (1990) |
| Acetate | -369.4 | -486.4 | 40.5 | Shock and Helgeson (1990) |
| Propionate | -363.29 | -513.43 | 54.95 | Shock and Helgeson (1990) |
| Butyrate | -354.25 | -535.62 | 70.30 | Shock and Helgeson (1990) |
| Glucose | -915.9 | -1262.2 | 112.2 | Amend and Plyasunov (2001) |
| Sulfate (SO_4_^2-^) | -744.96 | -910.21 | 13.88 | Shock et al. (1997) |
| Sulfide (HS^-^) | 11.97 | -16.12 | 20.65 | Shock et al. (1997) |

**Supplementary References**

Amend, J.P., and Plyasunov, A.V. (2001). Carbohydrates in thermophile metabolism: calculation of the standard molal thermodynamic properties of aqueous pentoses and hexoses at elevated temperatures and pressures. *Geochim. Cosmochim. Acta* 65**:**3901-3917.

Amend, J.P., and Shock, E.L. (2001). Energetics of overall metabolic reactions of thermophilic and hyperthermophilic Archaea and Bacteria. *FEMS Microbiol. Rev.* 25**:**175-243.

Fenn, W.O. (1971). Partial molar volumes of oxygen and carbon monoxide in blood. *Respiration Physiol.* 13**:**129-140.

Oelkers, E.H., Helgeson, H.C., Shock, E.L., Sverjensky, D.A., Johnson, J.W., and Pokrovskii, V.A. (1995). Summary of the Apparent Standard Partial Molal Gibbs Free Energies of Formation of Aqueous Species, Minerals, and Gases at Pressures 1 to 5000 Bars and Temperatures 25 to 1000 °C. *J. Phys. Chem. Ref. Data* 24**:**1401-1560.

Shock, E.L., and Helgeson, H.C. (1990). Calculation of the thermodynamic and transport properties of aqueous species at high pressures and temperatures: Standard partial molal properties of organic species. *Geochim. Cosmochim. Acta* 54**:**915-945.

Shock, E.L., Sassani, D.C., Willis, M., and Sverjensky, D.A. (1997). Inorganic species in geologic fluids: Correlations among standard molal thermodynamic properties of aqueous ions and hydroxide complexes. *Geochim. Cosmochim. Acta* 61**:**907-950.

Wagman, D.D., Evans, W.H., Parker, V.B., Schumm, R.H., Halow, I., Bailey, S.M., Churney, K.L., and Nuttall, R.L. (1982). NBS tables of chemical thermodynamic properties selected values for inorganic and C1 and C2 organic substances in SI units. *J. Phys. Chem. Ref. Data* 11.
