# Supplementary figures and images for: Origin of Short-Chain Organic Acids in Serpentinite Mud Volcanoes of the Mariana Convergent Margin
Source: Front Microbiol. 2019 Jul 26;10:1729. doi: 10.3389/fmicb.2019.01729 (PMC6677109; doi:10.3389/fmicb.2019.01729)

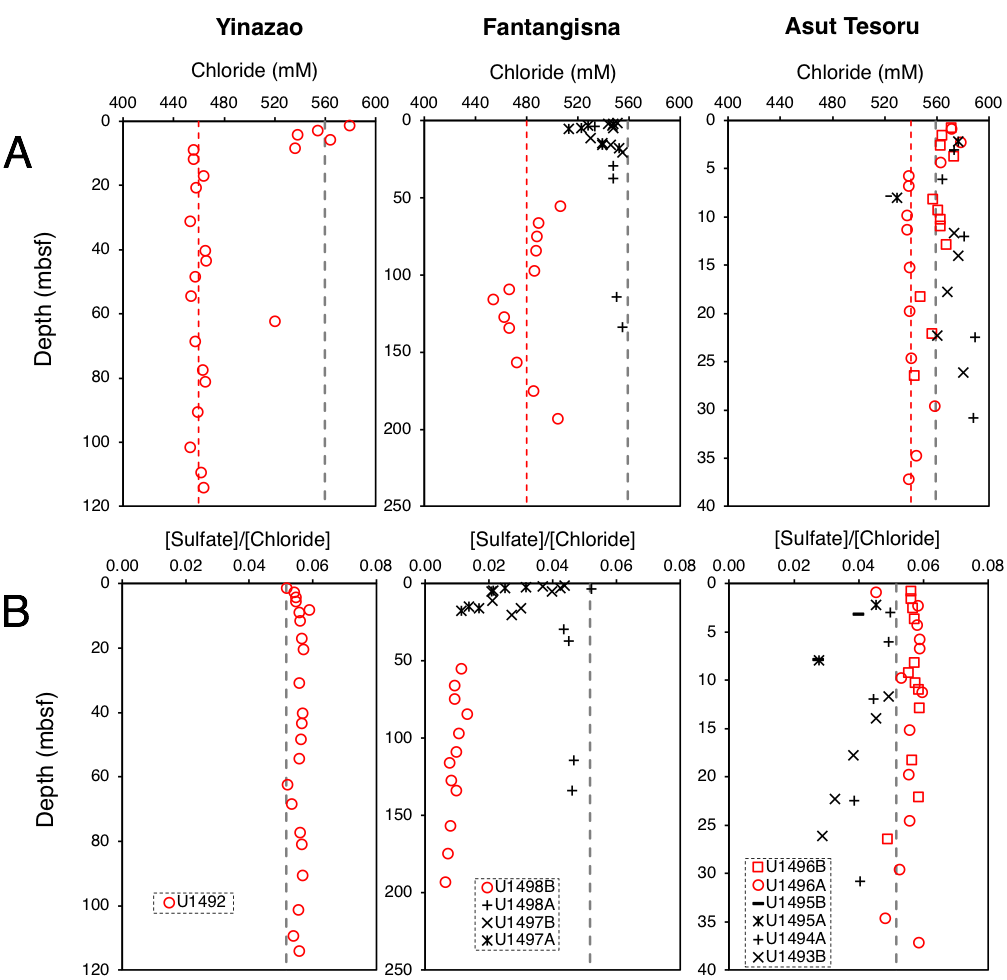

Supplement: Supplementary Figure S1 — (A) Chloride concentration depth profiles at all three mud volcanoes. (B) Depth profiles of sulfate-to-chloride concentration ratios (both mM). High-pH fluids (summit sites) are indicated by red marker symbols, moderate-pH fluids (flank sites) by black marker symbols. The dashed gray vertical lines indicate typical seawater chloride concentrations (559 mM) and seawater sulfate-to-chloride ratios [0.0517; assumes [sulfate] = 28.9 mM]. The dashed red vertical lines indicate chloride concentration asymptotes for high-pH fluids [Yinazao: 460 mM; Fantangisña: 480 mM; Asùt Tesoru (U1496A): 540 mM]. Chloride concentrations in high-pH fluids that are significantly below those in seawater indicate a potential freshwater contribution from dewatering of deeply buried clay minerals. [file Image_1.PNG]

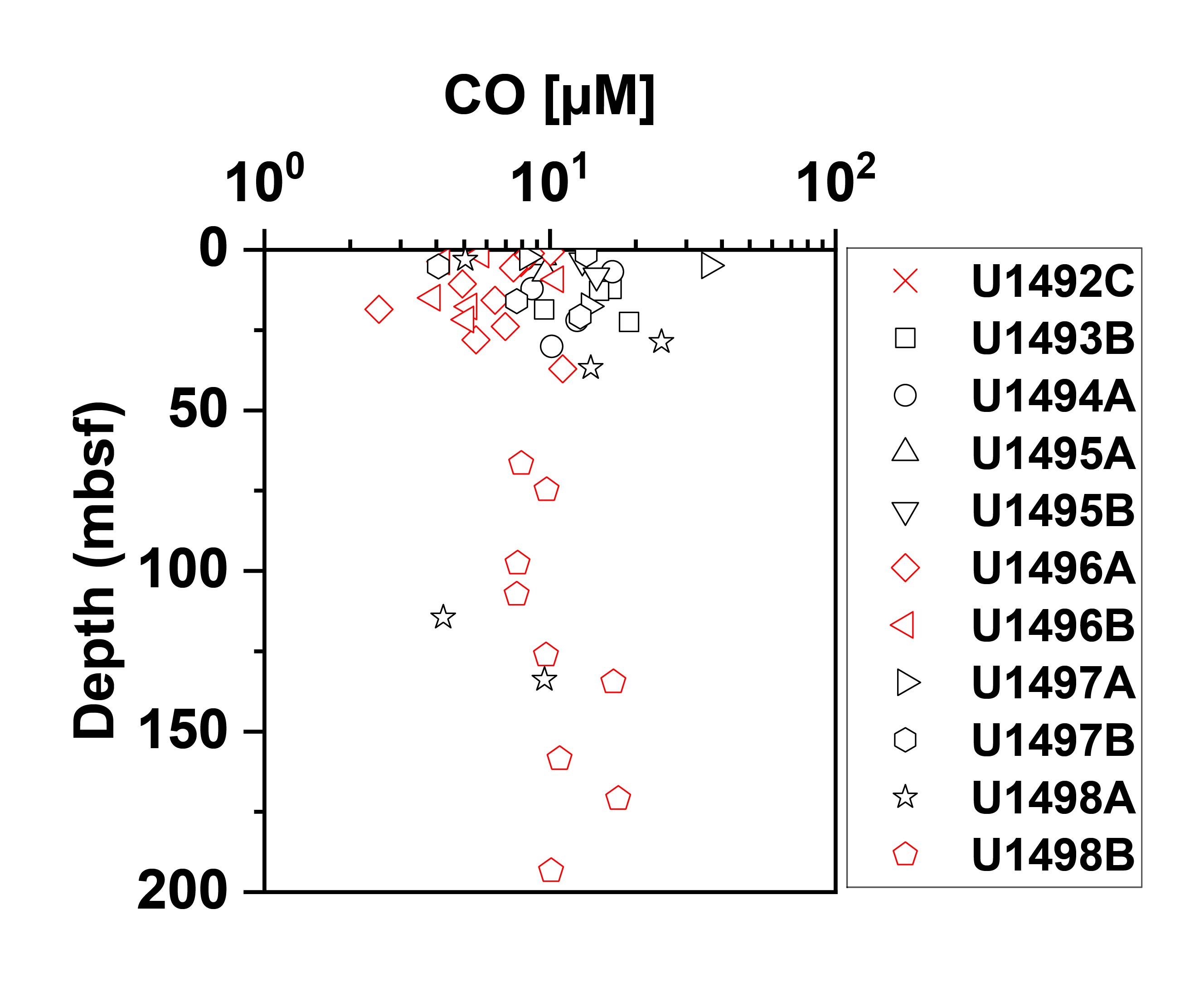

Supplement: Supplementary Figure S2 — Concentration profiles of pyruvate, lactate, and valerate across boreholes of the three mud volcanoes. [file Image_2.JPEG]

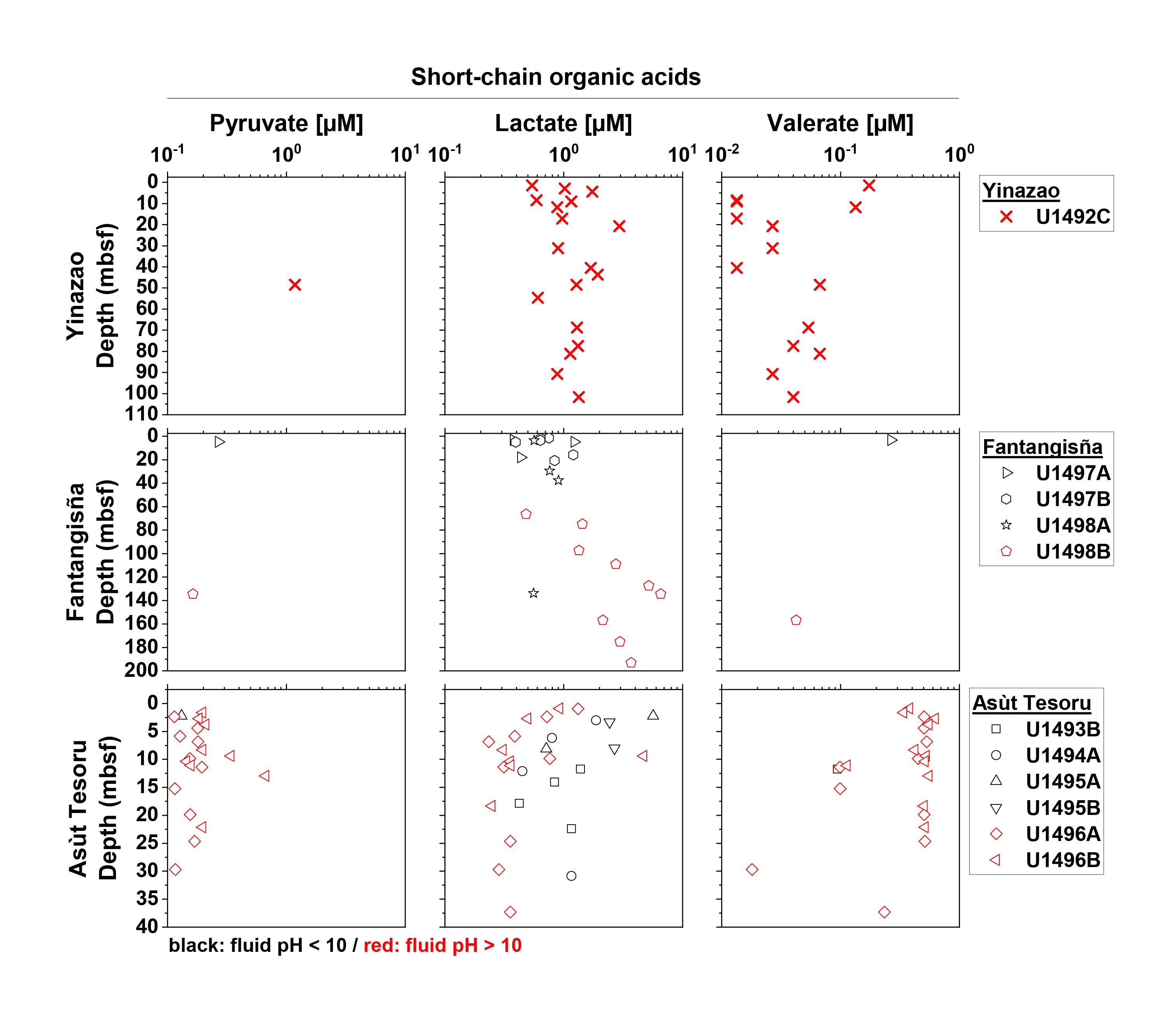

Supplement: Supplementary Figure S3 — Concentration profiles of CO across boreholes of the three mud volcanoes. [file Image_3.JPEG]

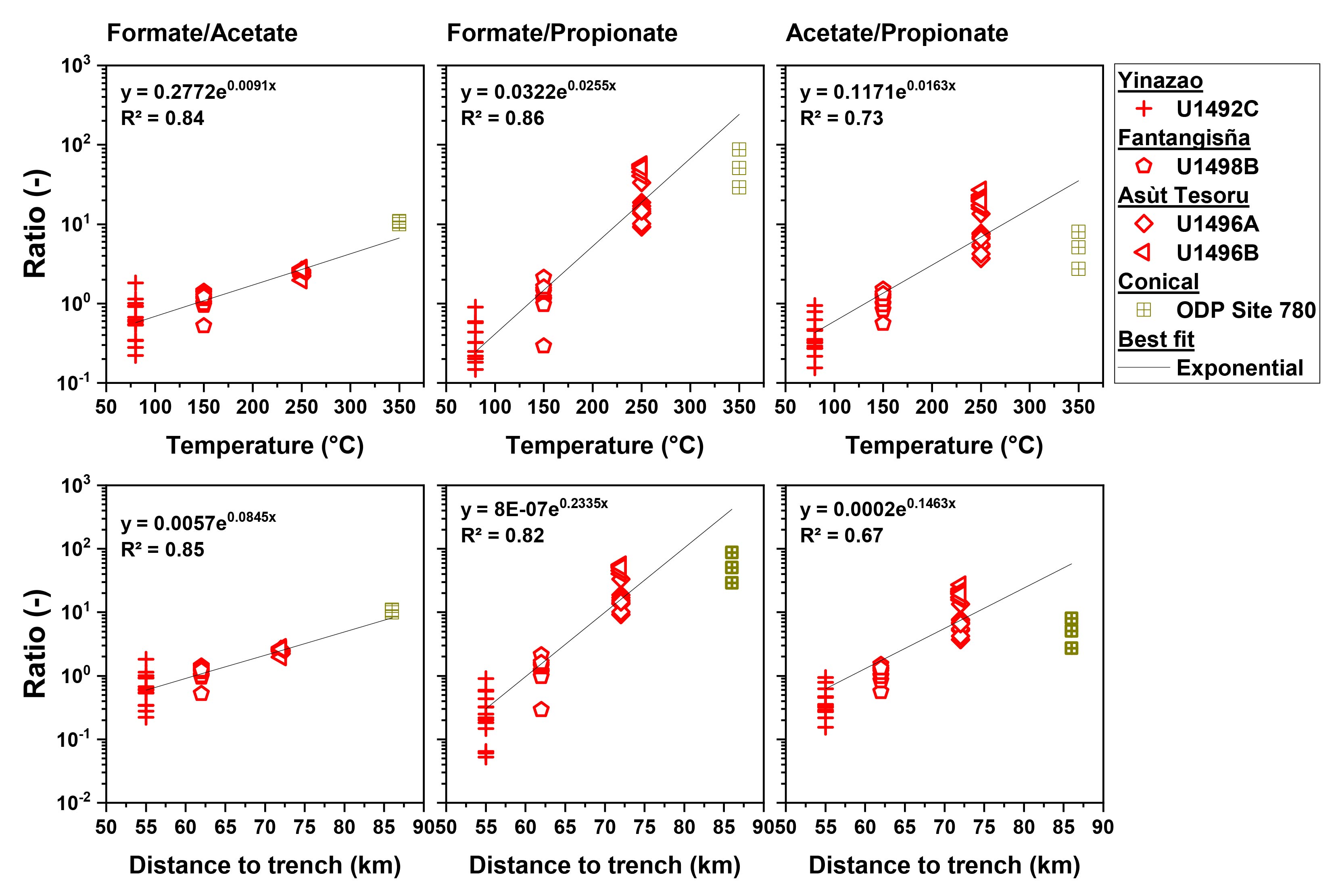

Supplement: Supplementary Figure S4 — Concentration ratios of formate-to-acetate, formate-to-propionate, and acetate-to-propionate vs. temperature in the subducting slab (upper panel) and distance to the Mariana Trench (lower panel). [file Image_4.JPEG]

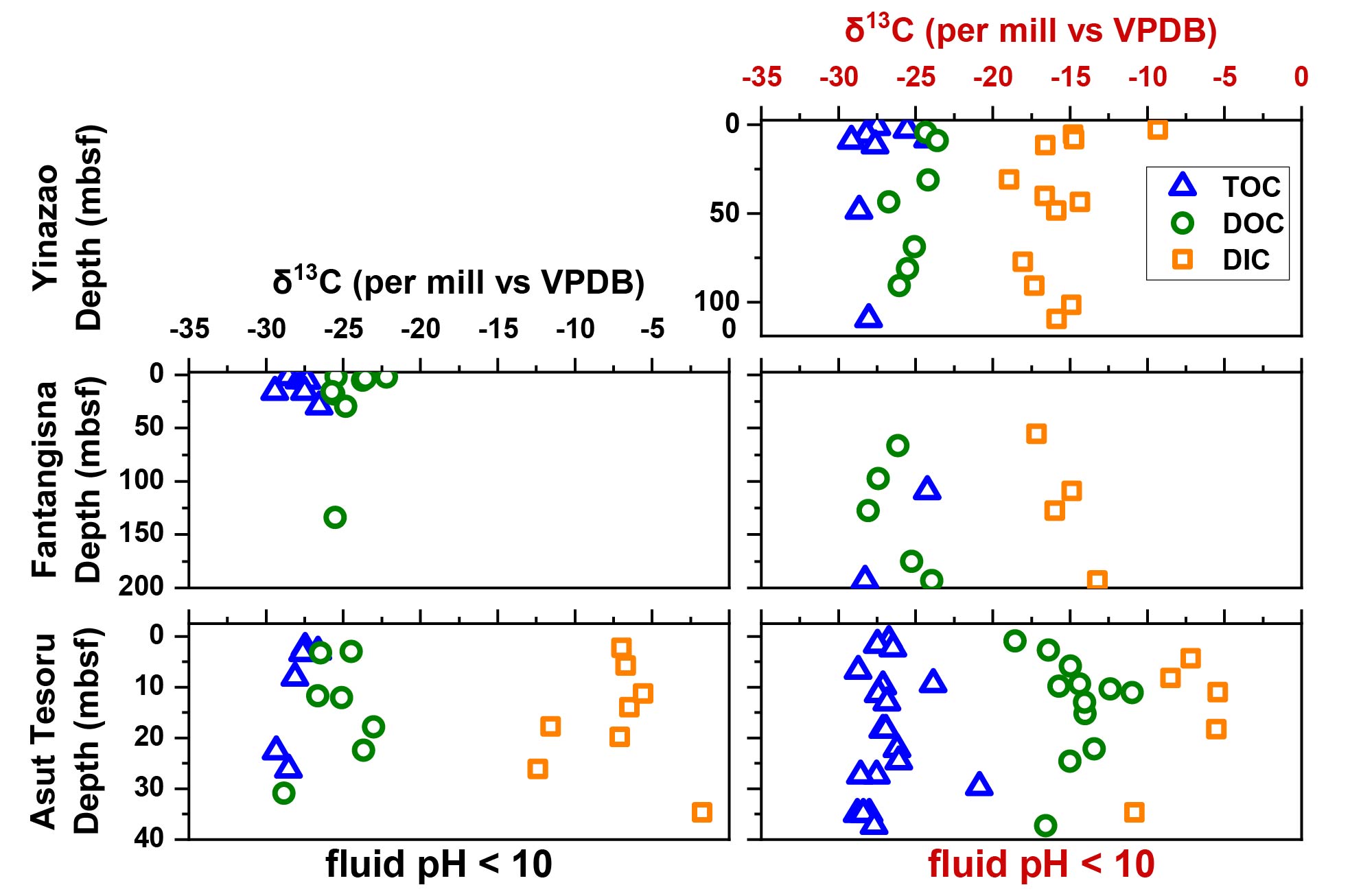

Supplement: Supplementary Figure S5 — δ13C-isotopic compositions of TOC, DOC, and DIC from moderate-pH (left column) and high-pH (right column) mud fluids of three serpentinite mud volcanoes drilled during IODP Expedition 366. [file Image_5.JPEG]
